# Synthesis of diindolylmethane (DIM) bearing thiadiazole derivatives as a potent urease inhibitor

**DOI:** 10.1038/s41598-020-64729-3

**Published:** 2020-05-14

**Authors:** Muhammad Taha, Fazal Rahim, Aftab Ahmad Khan, El Hassane Anouar, Naveed Ahmed, Syed Adnan Ali Shah, Mohamed Ibrahim, Zainul Amiruddin Zakari

**Affiliations:** 1 Department of clinical pharmacy, Institute for Research and Medical Consultations (IRMC), Imam Abdulrahman Bin Faisal University, P.O. Box 31441 Dammam, Saudi Arabia; 2 Department of Chemistry, Hazara University, Mansehra, 21300 Khyber Pakhtunkhwa, Pakistan; 3 Department of Chemistry, College of Science and Humanities in Al-Kharj, Prince Sattam bin Abdulaziz University, Al-Kharj, 11942 Saudi Arabia; 4 Department of Pharmacy, Quaid-i-Azam University, Islamabad, 45320 Pakistan; 5 Atta-ur-Rahman Institute for Natural Product Discovery (AuRIns), Universiti Teknologi MARA Cawangan Selangor Kampus Puncak Alam, 42300 Bandar Puncak Alam, Selangor Darul Ehsan, Malaysia, 42300 D. E., Selangor Malaysia; 6 Faculty of Pharmacy, Universiti Teknologi MARA Cawangan Selangor Kampus Puncak Alam, 42300 Bandar Puncak Alam, Selangor Darul Ehsan, Malaysia, 42300 Darul Ehsan, Selangor Malaysia; 7 Department of Biomedical Science, Faculty of Medicine and Health Sciences, Universiti Putra Malaysia, 43400 Serdang, Selangor Malaysia; 8 Halal Institute Research Institute, Universiti Putra Malaysia, 43400 Serdang, Selangor Malaysia

**Keywords:** Chemistry, Medicinal chemistry, Organic chemistry

## Abstract

The current study describes synthesis of diindolylmethane *(DIM) derivatives* based-thiadiazole as a new class of urease inhibitors. *Diindolylmethane* is natural product alkaloid reported to use in medicinal chemistry extensively. *Diindolylmethane-*based-thiadiazole analogs (**1–18**) were synthesized and characterized by various spectroscopic techniques ^1^HNMR, ^13^C-NMR, EI-MS and evaluated for urease (jack bean urease) inhibitory potential. All compounds showed excellent to moderate inhibitory potential having IC_50_ value within the range of 0.50 ± 0.01 to 33.20 ± 1.20 *µ*M compared with the standard thiourea (21.60 ± 0.70 *µ*M). Compound **8** (IC_50_ = 0.50 ± 0.01 *µ*M) was the most potent inhibitor amongst all derivatives. Structure-activity relationships have been established for all compounds. The key binding interactions of most active compounds with enzyme were confirmed through molecular docking studies.

## Introduction

Urease (EC 3.5.1.5) belongs to the family of amidohydrolase enzymes having two nickel atoms in their core structure. Urease involves the conversion of urea into ammonia and carbon dioxide or carboamate^1^. Among the superfamily of *bi*-nuclear metallohydrolases, urease is quite different from having Ni (II) ions in their active site. Urease is broadly found in nature and bio-synthesized by different organisms such as plants, fungi, bacteria, invertebrates, algae and are found in soil as soil enzyme^2,3^. Urease also plays a vital role in the germination process of plants and involved in nitrogen metabolism^4^. The fertilization of urea creates a major increase in the pH which is happened when the high amount is released resulting in increasing the alkalinity of soil which leads to high damage of plants by depriving them of their necessary nutrients^5,6^. Ureases plays an important role in most of the pathogenic processes in humans and animals. It plays a great role in peptic ulceration, pyelonephritis, arthritis, urolithiasis, kidney stone, urinary catheter and encephalopathy^7–9^.

The indole having great importance due to its unique chemical structure and a wide range of biological properties^10^. Mostly C-3-substituted indoles play an important role in many building blocks for the synthesis of different biologically active compounds having antimalarial^11^, inhibitors of HIV-1^12^, antimicrobial^13,14^, antileishmanial^15^, Urease inhibitors^16^, cytotoxic^17^, inhibitors of hepatitis C virus^18^, anti-diabetic^19^ and neuroprotective activities^20^. N-1 and C-3 substituted analogs of indole also showed anti-inflammatory, anti-nociceptive, anti-cancer, antioxidant and anti-psychotic activities^21–28^. Marine indole alkaloids have appeared as an important structural class showing anti-microbial, antitumor and anti-viral activity^29–31^. Some *bis*-Indole alkaloids with great biological importance have been collected from invertebrates such as bryozoans, tunicates, coelenterates, and sponges^32–35^. Five-membered nitrogen-containing heterocyclic compounds such as triazole and thiadiazole have great importance in medicinal chemistry because of their wide range of biological activities like antimicrobial, antifungal, antibacterial, antitumor, antiurease and antilipase^36–41^. Among different heterocycles, 1,2,4-triazole-3-thiones are important pharmacophores for urease inhibition because of their structural similarity to urea. Some 1,2,4-triazole derivatives were reported as potential urease inhibitors^42,43^.

**Figure 1:**
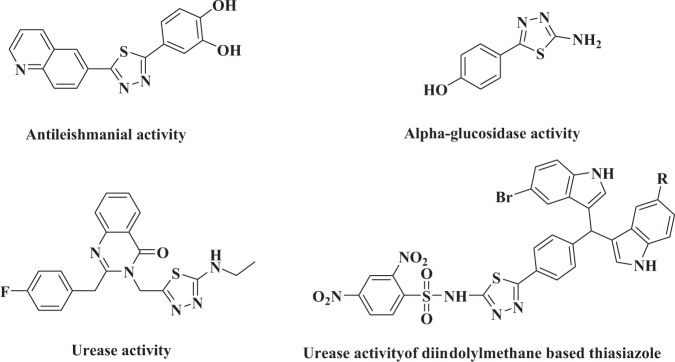
Rationalization of the newly synthesized diindolylmethane-based-thiadiazole analogs with already reported thiadiazole analogs.

Our research group have already reported thiazole, flavones, triazinoindole, isatin, benzimidazole, biscoumarin, and oxadiazole analogs as potential *α*-glucosidase inhibitors^44–53^. We have also reported thiadiazole analogs as potent antileishmanial, α-glucosidase and urease inhibitors shown in Fig. 1^54–56^. In this study, we have synthesized different substituted indole bearing thiadiazole analogs keeping in view their biological importance with the hope that it may show greater potential. Experimental results proved our previous hypothesis by obtaining good urease inhibitory potential of our synthesized molecules.

## Results and discussion

### Chemistry

The synthesis of diindolylmethane derivatives (**1-18**) was carried out in three steps. In the first step, 2 equivalents of indole were mixed with-4-formylbenzoic acid in acetic acid and reflux for 4–6 hours to afford intermediate product **I**. The Intermediate **I** was then treated with thiosemicarbazide in POCl_3_, then reflux for 3–4 hrs. to obtained intermediate **I**. The intermediate **II** was then mixed with different Aryl sulfonyl chloride to get the pure products **(1–18)** in high yield. All reactions completion was monitored by TLC.

### Urease activity

New series of diindolylmethane-based-thiadiazole analogs (**1–18**) synthesised and evaluated for their *in vitro* urease (jack bean urease) inhibitory activity. All the derivatives exhibited urease potential with IC_50_ value ranging between0.50 ± 0.01 to 33.20 ± 1.20 *µ*M as compared to the standard thiourea (21.60 ± 0.70 µM). Among the synthesised compounds, fifteen derivatives **8**, **6**, **3**, **9**, **10**, **2**, **4**, **7**, **15**, **13**, **12**, **1**, **14** and **18** with IC_50_ values 0.50 ± 0.01, 0.70 ± 0.01, 1.10 ± 0.01, 1.10 ± 0.01, 1.6 ± 0.01, 1.80 ± 0.01, 2.20 ± 0.10, 2.30 ± 0.10, 3.90 ± 0.10, 5.10 ± 0.10, 8.50 ± 0.30, 13.20 ± 0.30, 19.80 ± 0.80 and 20.40 ± 1.20 respectively, which is better than the standard thiourea.

The most active compound among the series is analog **8** (IC_50_ = 0.50 ± 0.01 *µ*M) having two-nitro groups at *ortho* and *para* position on phenyl ring. The greater inhibition shown by this compound is seemed due to electron-withdrawing group on phenyl ring. The second most active analog among the series is compound **6** (IC_50_ = 0.70 ± 0.01 *µ*M) having three chloro groups on phenyl ring at 2,4,5-position. The greater potential of this analog is also seeming due to having EWG at phenyl ring.

If we compare analog **8** (IC_50_ = 0.50 ± 0.01 *µ*M) with other nitro group containing analogs like **2**, a *para* nitro analog (IC_50_ = 1.80 ± 0.01 *µ*M) **3**, a *ortho* nitro analog **13**, (IC_50_ = 1.10 ± 0.01 *µ*M) *ortho* nitro *meta* methyl analog (IC_50_ = 5.10 ± 0.10 *µ*M) and **15**, a *ortho* nitro *meta methoxy* analog (IC_50_ = 3.90 ± 0.10 *µ*M), the compound **8** is superior. The difference in the inhibitory potential of all these analogs are seemed due to the difference in number and nature of substituents on phenyl ring.

The analog **7** (IC_50_ = 2.30 ± 0.10 *µ*M) and analog **10** (IC_50_ = 1.6 ± 0.01 *µ*M) having di-chloro groups at 2,4 and 3,4 position respectively when compare with analog **6** (IC_50_ = 0.70 ± 0.01 *µ*M) having tri-chloro groups shows more potency as compared to analog **7** and **10**. The difference in their activity seems due to substituents different numbers and positions on phenyl ring.

Similarly the decline in inhibition was observed when EWG is replaced by methyl group as shown in analog **16** (IC_50_ = 23.80 ± 1.00) having methyl at *the ortho* position of phenyl ring with analog **17** (IC_50_ = 28.60 ± 1.20) having methyl at *meta* position and analog **18** (IC_50_ = 20.40 ± 1.20) having methyl at *the para* position. All of the three analogs contain methyl groups attached at different positions showed a different kind of inhibition, which might be due to attachment of substituents at a different position on phenyl ring. In the current study, we have found that inhibitory potential was greatly affected by the nature, position, and number of substituents. All those analogs having electron-withdrawing groups (EWG) on phenyl ring showed greater potential as compared to those analogs having electron-donating groups (EDG). The binding interaction was confirmed through molecular docking studies.

### Molecular docking

The IC_50_ values diindolylmethane bearing thiadiazol derivatives as a potent urease inhibitor are presented in Table 1. The urease inhibition by the synthesized derivatives may strongly related to the type, number, positions of the functional group in the aromatic ring of basic skeleton of diindolylmethane bearing thiadiazol derivatives and to the strength of the intermolecular interaction that may have formed these functional groups and the residues of the active of urease (Table 1). To understand the urease inhibition by the synthesized derivatives, a molecular docking study has been carried out to determine the binding modes of all synthesized derivatives **1–18** from one side and the active residues of the urease from another side. These compounds differ by the number and position of the substituted functional groups in the aromatic ring (Table 1). For instance, compounds **2, 3** and **10** are substituted by a mono nitro in the group in *para* and *ortho* positions, and di-nitro groups in *ortho* and *para* positions, respectively (Table 1). Compounds **6**, **7** and **10** also differ by the number and positions of substituted chloro groups (Table 1). **16–18** are monosubstituted by a methyl group at *ortho*, *meta* and *para* positions respectively (Table 1). Table 2 summarized the calculated binding energies of the stable complexes ligand-urease, the number of established intermolecular hydrogen bonding between the synthesized compounds (**1–18**) and active site residues of urease.

**Table 1:** Different diindolylmethane-based-thiadiazole analogs and their urease activity (1–18).

S.No.	R	IC_50_ ± SEM^a^	S.No.	R	IC_50_ ± SEM^a^
**1**		13.20 ± 0.30	**10**		1.6 ± 0.01
**2**		1.80 ± 0.01	**11**		33.20 ± 1.20
**3**		1.10 ± 0.01	**12**		8.50 ± 0.30
**4**		2.20 ± 0.10	**13**		5.10 ± 0.10
**5**		7.30 ± 0.10	**14**		19.80 ± 0.80
**6**		0.70 ± 0.01	**15**		3.90 ± 0.10
**7**		2.30 ± 0.10	**16**		23.80 ± 1.00
**8**		0.50 ± 0.01	**17**		28.60 ± 1.20
**9**		1.10 ± 0.01	**18**		20.40 ± 1.20
**Standard Drug**		**Thiourea**		**21.60** ± **0.70**

**Table 2:** IC_50_, docking binding energies, hydrogen bonding and the number of closest residues to the docked ligands in the active site of the diindolylmethane bearing thiadiazol derivatives **1–18** into the active binding site of urease.

No. of Compound	Free binding energy (kcal/mol)	H-Bonds (HBs)	Number of closest residues to the docked ligand in the active site	IC_50_ ± SEM
**2**	−8.19	4	9	1.80 ± 0.01
**3**	−8.70	4	12	1.10 ± 0.01
**8**	−8.72	2	10	0.50 ± 0.01
**4**	−9.64	4	12	2.20 ± 0.10
**5**	−10.18	3	13	7.30 ± 0.10
**15**	−9.87	3	13	3.90 ± 0.10
**12**	−10.00	2	12	8.50 ± 0.30
**13**	−9.74	4	12	5.10 ± 0.10
**6**	−9.12	2	12	0.70 ± 0.01
**7**	−9.01	0	13	2.30 ± 0.10
**10**	−9.45	3	11	1.6 ± 0.01
**1**	−8.70	3	11	13.20 ± 0.30
**11**	−10.55	2	14	33.20 ± 1.20
**16**	−9.48	2	13	23.80 ± 1.00
**17**	−10.13	3	12	28.60 ± 1.20
**18**	−9.74	3	11	20.40 ± 1.20

**Figure 2:**
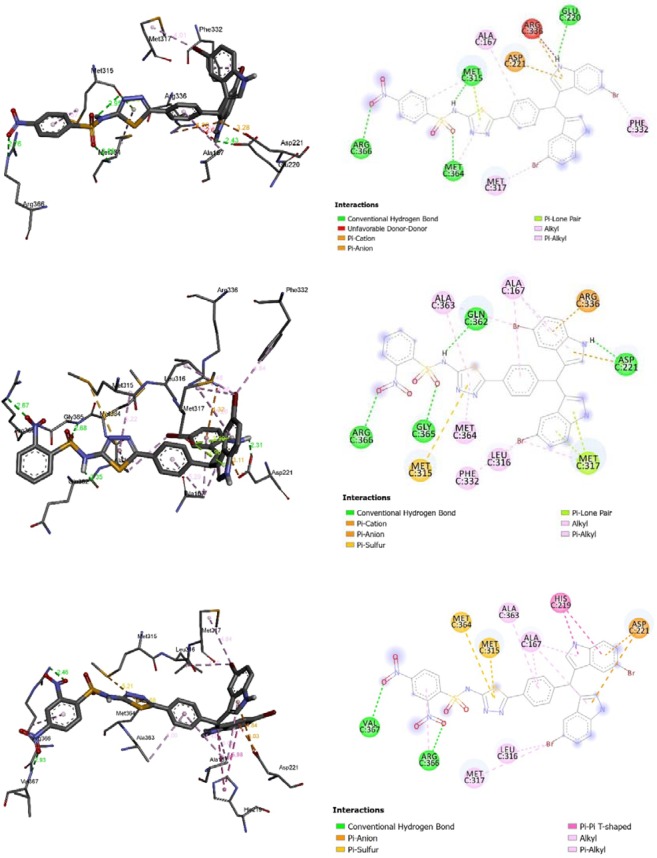
3D (right) and 2D (left) closest interactions between active site residues of urease and selected compounds **2**, **3**, and **8**.

The formed complexes between diindolylmethane bearing thiadiazol derivatives (**1–18**) and the active residues of urease displayed negative bending energies, which indicates that the inhibition of urease by the selected diindolylmethane bearing thiadiazol derivatives is thermodynamically favorable (Table 2). From the docking results in Table 2, binding energies of the stable complexes vary slightly from −10.55 to −8.20 kcal/mol. Such variation is low enough to be considered as a potent descriptor in rationalizing the observed inhibition of urease by the selected derivatives. However, the number of hydrogen bonding, its distances and intermolecular interactions between the substitute groups of the selected derivatives and the active residues may strongly help in understanding the observed urease inhibition by these selected compounds. For instance, the higher urease inhibition of **8** compared with **2** and **3** may refer to the number of hydrogen bonding formed between the nitro groups in the former and latter (Fig. 2). Indeed, in the **8**-urease complex two hydrogen bonds are formed between the nitro groups at ortho and para positions with ARG 366 and VAL 367 amino acids of distances 2.46 and 2.93 Å, respectively. While in **2**-urease and **3**-urease complexes, the hydrogen bonds are formed between the nitro group at *para* (**2**) and *ortho* (**3**) positions with ARG 336 amino acid of distances 2.76 and 2.67 Å, respectively. The higher urease inhibition of **3** compared with **2** may also refer to the stronger hydrogen bond formed with the former (2.76Å) compared with the latter (2.67 Å).

Similarly, the higher urease inhibition of **6** compared with **7** and **10** may refer to the number of residues that interact with chloro groups in the former and to the strength of these interactions (Table 2).

**Figure 3:**
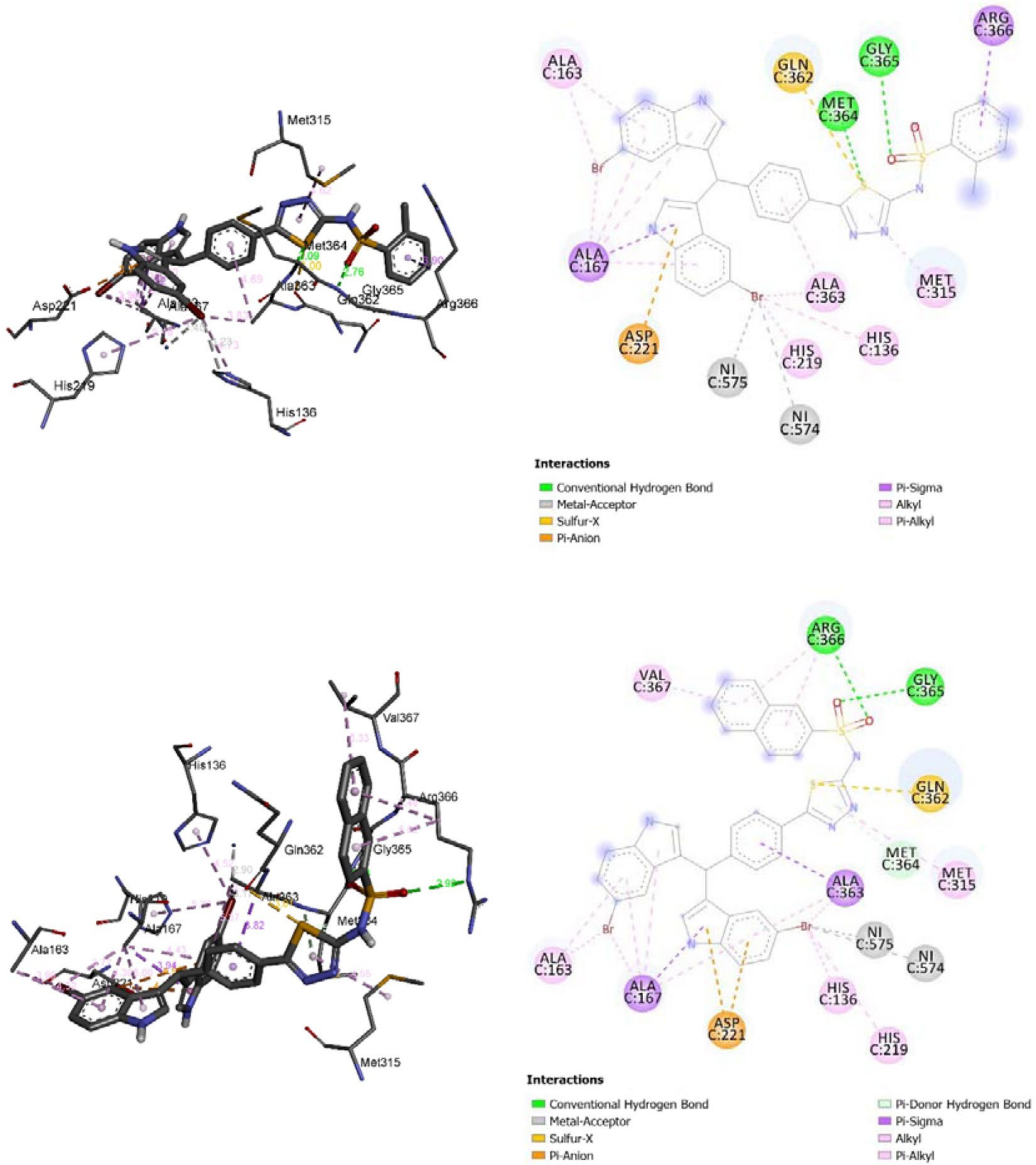
3D (right) and 2D (left) closest interactions between active site residues of urease and compounds **16** and **11**.

The diindolylmethane bearing thiadiazol derivatives monosubstituted with chlorine (**6–7**,**10**), nitro (**2–3**,**8**), or disubstituted with functional groups (chlorine, nitro, hydroxyl, methoxy, and bromine) showed higher urease inhibition than those monosubstituted with methyl (**16**–**18**) and benzene ring (**11**). The significant decrease of urease inhibition in **16**–**18** and **11** may refer to the fact that these groups are not involved in intermolecular interactions with the closest residues of urease (**16**–1**8**) or too weak interactions in case of **11** (Fig. 3).

## Conclusion

We synthesized eighteen analogs (**1–18**) of diindolylmethane-based-thiadiazole (**1–18**) and evaluated against urease inhibitory potential. All analogs showed excellent to a good inhibitory potential having IC_50_ ranging from IC_50_ = 0.50 ± 0.01 to 33.20 ± 1.20 µM) as compared to the standard thiourea (21.60 ± 0.70 µM). Analog **8** (IC_50_ value 0.50 ± 0.01 *µ*M) is the most potent among the series. The structure-activity relationship was mainly based upon by bringing about the difference of substituents on phenyl rings. For binding interaction of the most active analogs molecular docking study was performed.

## Experimental

### Material and methods

NMR experiment was carried out on Avance Bruker AM 500 MHz (Wissembourg, Switzerland). TLC was done on pre-coated silica gel aluminum plates (Kieselgel 60, 254, E. Merck, Darmstadt, Germany). Chromatograms were envisioned through UV at 254 and 365 nm.

### Synthesis of 4-(bis(5-bromo-1H-indol-3-yl)methyl)benzoic acid (I)

A mixture of 5-bromo-1H-indole (9.75 g, 50 mmol), 4-formylbenzoic acid (3.75 g, 25 mmol) and a catalytic amount of acetic acid in methanol (50 mL) was heated under reflux for 6 hours.

The mixture was dried and the crude product (**I**) was washed with diethyl ether, then crystallized from methanol and gives pinkish solid, (11.2 g, 90.0%); R_ƒ._ 0.46 (ethylecetate/hexane 4:6); m.p. 288–289 °C; IR (KBr): 3530–2570 cm^−1^ br. (COOH-str), 1680 cm^−1^ (C=0 str), 1617 cm^−1^ (Ar C=C), 1146 cm^−1^ (C-O str), 630 cm^−1^ (C-Br str); ^1^H NMR (500 MHz, DMSO-d_6_): δ 11.90 (s, 2H, NH), 7.92–7.86 (m, 4H), 7.72 (t, *J* = 7.8 Hz, 2H), 7.44 (d, *J* = 7.7 Hz, 2H), 7.40 (s, 2H), 7.32 (td, *J* = 7.5, 2.0 Hz, 2H), 5.73 (s, 1H), 4.08 (s, 1H, OH); ^13^C-NMR (150 MHz, DMSO-d6,): *δ* 169.3, 143.1, 135.2, 135.2, 130.1, 130.1, 129.5, 129.5, 128.9, 128.9, 126.9, 122.7, 122.7, 121.0, 121.0, 120.9, 120.9, 120.7, 120.7, 117.1, 117.1, 113.2, 113.2, 55.1; HREI-MS: m/z calcd for C_24_H_16_Br_2_N_2_O_2_ [M + 4]^+^ 525.9520, [M + 3]^+^ 524.9580, [M + 2]^+^ 523.9548, [M + 1]^+^ 522.9605, [M]^+^ 521.9560.

### Synthesis of 5-(4-(bis(5-bromo-1H-indol-3-yl)methyl)phenyl)-1,3,4-thiadiazol-2-amine

The 4-(bis(5-bromo-1H-indol-3-yl)methyl)benzoic acid (20 mmol) was heated under reflux with thiosemicarbazide (21mmole) in POCl_3_ for 6 hours. The completion of reaction was monitored by TLC. The mixture of reaction was poured in cold water. The precipitate formed was washed with dilute sodium bicarbonate solutions and recrystallized in ethanol to get pure compound (**II**).

Yellow solid (11.2 g, 90.0%); R_ƒ._ 0.60 (ethylecetate/hexane 4:6); m.p. 288–289 °C; IR (KBr): 3420 cm^−1^ (NH-str), 3230 cm^−1^ (2°amine N-H Str), 1615 cm^−1^ (Ar C=C), 1351 cm^−1^ (N-S=O), 626 cm^−1^ (C-Br str); ^1^H NMR (500 MHz, DMSO-d_6_): δ 11.96 (s, 2H, NH), 7.90–7.85 (m, 4H), 7.71 (t, J = 7.6 Hz, 2H), 7.43 (d, *J* = 7.5 Hz, 2H), 7.42 (s, 2H), 7.31 (t, *J* = 7.7 Hz, 2H), 5.72 (s, 1H), 4.26 (s, 2H, NH_2_); ^13^C-NMR (150 MHz, DMSO-d6,): *δ* 175.3, 161.2, 143.2, 135.3, 135.3, 130.2, 130.2, 129.4, 129.4, 128.8, 128.8, 126.8, 122.6, 122.6, 121.2, 121.2, 120.8, 120.8, 120.6, 120.6, 117.2, 117.2, 113.1, 113.1, 55.3; HREI-MS: m/z calcd for C_25_H_17_Br_2_N_5_S [M + 4]^+^ 580.9520, [M + 3]^+^ 579.9575, [M + 2]^+^ 578.9542, [M + 1]^+^ 577.9601, [M]^+^ 576.9553.

### General procedure for the synthesis of diindolylmethane-based-thiadiazole analogs Characterization (1–18)

The intermediate (**II**) was treated with different aryl sulfonyl chloride in the presence of pyridine under stirring for overnight. After completion of reaction the solvent was removed and the crude product was washed with cold water, filtered and dried. The crude product was recrystallized from ethanol. NMR spectra of all **1-18** compounds are provided in supplementary data.

#### *N*-(5-(4-(bis(5-bromo-1H-indol-3-yl)methyl)phenyl)-1,3,4-thiadiazol-2-yl)benzenesulfonamide (1)

Orange solid. Yield: 81.40%; R_ƒ._ 0.70 (ethylecetate/hexane 3:7); m.p.: 301–302 °C; IR (KBr): 3255 cm^−1^ (2°amine N-H Str), 1612 cm^−1^ (Ar C=C), 1360 cm^−1^ (N-S=O), 638 cm^−1^ (C-Br str); 1H NMR (500 MHz, DMSO-d6): *δ* 11.98 (s, 1H, NH), 11.52 (s, 1H, NH), 11.17 (s, 1H, NH), 7.81 (d, *J* = 7.4 Hz, 2H), 7.50 (t, *J* = 7.2 Hz, 2H), 7.41 (d, *J* = 7.2 Hz, 2H), 7.40 (d, *J* = 8.4 Hz, 2H), 7.31–7.27 (m, 3H), 7.11 (t, *J* = 7.2 Hz, 2H), 6.94 (t, *J* = 7.2 Hz, 2H), 6.85 (s, 2H), 5.98 (s, 1H, CH);^13^C-NMR (125 MHz, DMSO-*d*6): *δ*174.1, 173.0, 139.7, 138.1, 135.5, 135.5, 131.9, 130.5, 129.6, 129.6, 129.0, 129.0, 129.5, 129.5, 127.4, 127.4, 127.3, 127.3, 124.7, 124.7, 123.0, 123.0, 121.0, 121.0, 116.4, 116.4, 113.2, 113.2, 112.1, 112.1, 54.6: HREI-MS: m/z Calcd for C_31_H_21_Br_2_N_5_O_2_S_2_ [M + 4]^+^ 720.9455, [M + 3]^+^ 719.9511, [M + 2]^+^ 718.9477, [M + 1]^+^ 717.9530, [M]^+^ 716.9496.

#### *N*-(5-(4-(bis(5-bromo-1H-indol-3-yl)methyl)phenyl)-1,3,4-thiadiazol-2-yl)-4-nitrobenzenesulfonamide (2)

Yellow. Yield: 80.0%; R_ƒ._ 0.56 (ethylecetate/hexane 3:7); m.p.: 312–313 °C; IR (KBr): 3242 cm^−1^ (2°amine N-H Str), 1625 cm^−1^ (Ar C=C), 1556 cm^−1^ (N-O str), 1355 cm^−1^ (N-S=O), 630 cm^−1^ (C-Br str); ^1^H NMR (500 MHz, DMSO-d_6_): *δ*12.42 (s, 1H, NH), 12.01 (s, 1H, NH), 10.98 (s, 1H, NH), 8.01 (d, *J* = 7.7 Hz, 2H), 7.93 (d, *J* = 7.7 Hz, 2H), 7.52 (d, *J* = 8.2 Hz, 2H), 7.41 (d, *J* = 8.2 Hz, 2H), 7.32 (d, *J* = 7.6 Hz, 2H), 7.13 (t, *J* = 7.4 Hz, 2H), 6.91 (t, *J* = 7.4 Hz, 2H), 6.86 (s, 2H), 5.98 (s, 1H); ^13^C-NMR (125 MHz, DMSO-*d*6): *δ*174.1, 173.0, 151.1, 146.3, 145.8, 142.3, 136.8, 136.5, 135.2, 131.8, 130.5, 130.3, 129.5, 129.5, 128.2, 128.2, 127.4, 127.4, 127.4, 124.2, 124.2, 121.7, 121.7, 120.0, 119.8, 118.0, 111.2, 111.1, 47.3, 13.3: HREI-MS: m/z Calcd for C_31_H_20_Br_2_N_6_O_4_S_2_ [M + 4]^+^ 765.9302, [M + 3]^+^ 764.9352, [M + 2]^+^ 763.9321, [M + 1]^+^ 762.9379, [M]^+^ 761.9342.

#### *N*-(5-(4-(bis(5-bromo-1H-indol-3-yl)methyl)phenyl)-1,3,4-thiadiazol-2-yl)-2-nitrobenzenesulfonamide (3)

Light yellow, Yield: 78.0%; R_ƒ._ 0.61 (ethylecetate/hexane 3:7); m.p.: 314–315 °C; IR (KBr): 3246 cm^−1^ (2°amine N-H Str), 1621 cm^−1^ (Ar C=C), 1552 cm^−1^ (N-O str), 1349 cm^−1^ (N-S=O), 632 cm^−1^ (C-Br str); ^1^H NMR (500 MHz, DMSO-d_6_): *δ*12.09 (s, 1H, NH), 11.32 (s, 1H, NH), 8.72 (s, 1H, NH), 8.11 (d, *J* = 7.6 Hz, 2H), 7.92 (t, *J* = 8.2 Hz, 2H), 7.82 (t, *J* = 7.5 Hz, 2H), 7.68–7.64 (m, 2H), 7.51 (d, *J* = 8.3 Hz, 2H), 7.38 (d, *J* = 8.3 Hz, 2H), 7.32 (d, *J* = 7.7 Hz, 2H), 6.94 (t, *J* = 7.1 Hz, 2H), 6.87 (s, 2H), 5.98 (s, 1H);^13^C-NMR (125 MHz, DMSO-*d*6): *δ*174.1, 173.0, 147.2, 138.1, 135.5, 135.5, 135.1, 134.4, 132.8, 130.5, 129.6, 129.6, 129.5, 129.5, 128.2, 127.4, 127.4, 124.7, 124.7, 124.2, 123.0, 123.0, 121.0, 121.0, 116.4, 116.4, 113.2, 113.2, 112.1, 112.1, 54.6: HREI-MS: m/z Calcd for C_31_H_20_Br_2_N_6_O_4_S_2_ [M + 4]^+^ 765.9306, [M + 3]^+^ 764.9355, [M + 2]^+^ 763.9324, [M + 1]^+^ 762.9373, [M]^+^ 761.9347.

#### *N*-(5-(4-(bis(5-bromo-1H-indol-3-yl)methyl)phenyl)-1,3,4-thiadiazol-2-yl)-2-hydroxy-5-methoxybenzenesulfonamide (4)

Orange solid. Yield: 82.0%; R_ƒ._ 0.55 (ethylecetate/hexane 3:7); m.p.: 298–297 °C; IR (KBr): 3470 cm^−1^ (OH-str), 3210 cm^−1^ (2°amine N-H Str), 1345 cm^−1^ (N-S=O), 1148 cm^−1^ (C-O str), 624 cm^−1^ (C-Br str); ^1^H NMR (500 MHz, DMSO-d_6_): 11.88 (s, 1H, NH), 10.52 (s, 1H, NH), 9.58 (s, 1H, NH), 9.21(s, 1H, OH), 7.80 (d, *J* = 8.1 Hz, 2H), 7.44 (d, *J* = 8.1 Hz, 2H), 7.37 (d, *J* = 8.1 Hz, 2H), 7.31 (d, *J* = 7.4 Hz, 2H), 7.11 (t, *J* = 7.4 Hz, 2H), 6.93–6.90 (m, 3H), 6.78 (d, *J* = 8.3 Hz, 2H), 5.96 (s, 1H), 3.80 (s, 3H, CH_3_); ^13^C-NMR (125 MHz, DMSO-*d*6): *δ*174.1, 173.0, 152.9, 151.9, 138.1, 135.5, 135.5, 130.5, 129.6, 129.6, 129.5, 129.5, 127.4, 127.4, 124.7, 124.7, 123.0, 123.0, 122.2, 121.0, 121.0, 121.0, 118.9, 116.4, 116.4, 113.2, 113.2, 112.6, 112.1, 112.1, 55.8, 54.6: HREI-MS: m/z Calcd for C_32_H_23_Br_2_N_5_O_4_S_2_ [M + 4]^+^ 766.9510, [M + 3]^+^ 765.9562, [M + 2]^+^ 764.9530, [M + 1]^+^ 763.9582, [M]^+^ 762.952.

#### N-(5-(4-(bis(5-bromo-1H-indol-3-yl)methyl)phenyl)-1,3,4-thiadiazol-2-yl)-5-chloro-2-methoxybenzenesulfonamide (5)

Organge solid. Yield: 80.0%; R_ƒ._ 0.68 (ethylecetate/hexane 3:7); m.p.: 315–316 °C; IR (KBr): 3235 cm^−1^ (2°amine N-H Str), 1619 cm^−1^ (Ar C=C), 1350 cm^−1^ (N-S=O), 1122 cm^−1^ (C-O str), 843 cm^−1^ (C-Cl str), 616 cm^−1^ (C-Br str); ^1^H NMR (500 MHz, DMSO-d_6_): *δ*11.84(s, 2H, NH), 10.98 (s, 1H, NH), 7.80 (d, *J* = 7.5 Hz, 2H), 7.63 (d, *J* = 7.6 Hz, 1H), 7.51–7.45 (m, 2H), 7.38 (d, *J* = 8.3 Hz, 2H), 7.31(d, *J* = 7.7 Hz, 2H), 7.13 (t, *J* = 7.4 Hz, 2H), 6.93 (t, *J* = 7.4 Hz, 2H), 6.86 (s, 2H), 5.97 (s, 1H), 3.75 (s, 3H, OCH_3_); ^13^C-NMR (125 MHz, DMSO-*d*6): *δ*174.1, 173.0, 155.8, 138.1, 135.5, 135.5, 134.1, 130.5, 129.6, 129.6, 129.5, 129.5, 127.6, 127.4, 127.4, 126.9, 124.7, 124.7, 123.0, 123.0, 121.0, 121.0, 121.0, 116.4, 116.4, 116.0, 113.2, 113.2, 112.1, 112.1, 55.8, 54.6: HREI-MS: m/z Calcd for C_32_H_22_Br_2_ClN_5_O_3_S_2_ [M + 4]^+^ 784.9165, [M + 3]^+^ 783.9225, [M + 2]^+^ 782.9186, [M + 1]^+^ 781.9242, [M]^+^ 780.9205.

#### N-(5-(4-(bis(5-bromo-1H-indol-3-yl)methyl)phenyl)-1,3,4-thiadiazol-2-yl)-2,4,5-trichlorobenzenesulfonamide (6)

Brown. Yield: 78.0%; R_ƒ._ 0.70 (ethylecetate/hexane 3:7); m.p.: 299–300 °C; IR (KBr): 3231 cm^−1^ (2°amine N-H Str), 1630 cm^−1^ (Ar C=C), 1356 cm^−1^ (N-S=O), 820 cm^−1^ (C-Cl str), 621 cm^−1^ (C-Br str); ^1^H NMR (500 MHz, DMSO-d_6_): *δ*11.71(s, 1H, NH), 10.68 (s, 1H, NH), 9.53 (s, 1H, NH),7.80 (d, *J* = 8.4 Hz, 2H), 7.50 (d, *J* = 8.4 Hz, 2H), 7.40 (d, *J* = 8.2 Hz, 2H), 7.33 (d, *J* = 7.4 Hz, 2H), 6.91 (t, *J* = 7.2 Hz, 2H), 6.85 (s, 2H), 6.81 (s, 1H), 6.30 (s, 1H), 5.96 (s, 1H); ^13^C-NMR (125 MHz, DMSO-*d*6): *δ*174.1, 173.0, 139.2, 138.1, 138.0, 135.5, 135.5, 132.1, 131.8, 131.0, 130.5, 129.6, 129.6, 129.5, 129.5, 129.4, 127.4, 127.4, 124.7, 124.7, 123.0, 123.0, 121.0, 121.0, 116.4, 116.4, 113.2, 113.2, 112.1, 112.1, 54.6: HREI-MS: m/z Calcd for C_31_H_18_Br_2_Cl_3_N_5_O_2_S_2_ [M + 6]^+^ 824.8246, [M + 5]^+^ 823.8316, [M + 4]^+^ 822.8288, [M + 3]^+^ 821.8332, [M + 2]^+^ 820.8306,[M + 1]^+^ 819.8358 [M + ]^+^ 818.8320.

#### N-(5-(4-(bis(5-bromo-1H-indol-3-yl)methyl)phenyl)-1,3,4-thiadiazol-2-yl)-2,4-dichlorobenzenesulfonamide (7)

Brown solid. Yield: 76.0%; R_ƒ._ 0.71 (ethylecetate/hexane 3:7); m.p.: 296–295 °C IR (KBr): 3224 cm^−1^ (2°amine N-H Str), 1624 cm^−1^ (Ar C=C), 1364 cm^−1^ (N-S=O), 790 cm^−1^ (C-Cl str), 643 cm^−1^ (C-Br str); ^1^H NMR (500 MHz, DMSO-d_6_): *δ* 11.79(s, 1H, NH), 11.52 (s, 1H, NH), 9.88 (s, 1H, NH), 7.80 (d, *J* = 7.4 Hz, 2H), 7.49 (d, *J* = 8.3 Hz, 2H), 7.38 (d, *J* = 8.1 Hz, 2H), 7.30 (d, *J* = 7.7 Hz, 2H), 7.26 (d, *J* = 8.3 Hz, 1H), 6.92 (t, J = 7.2 Hz, 2H), 6.85 (s, 2H), 6.32 (d, *J* = 8.1 Hz, 1H), 6.31 (s, 1H), 5.97 (s, 1H); ^13^C-NMR (125 MHz, DMSO-*d*6): *δ*174.1, 173.0, 138.9, 138.1, 137.8, 135.5, 135.5, 132.9, 130.7, 130.5, 130.1, 129.6, 129.6, 129.5, 129.5, 127.4, 127.2, 124.7, 124.7, 127.4, 123.0, 123.0, 121.0, 121.0, 116.4, 116.4, 113.2, 113.2, 112.1, 112.1, 54.6: HREI-MS: m/z Calcd for C_31_H_19_Br_2_Cl_2_N_5_O_2_S_2_ [M + 6]^+^ 790.8644, [M + 5]^+^ 789.8701, [M + 4]^+^ 788.8661, [M + 3]^+^ 787.8722, [M + 2]^+^ 786.8698, [M + 1]^+^ 785.8742 [M + ]^+^ 784.8718.

#### N-(5-(4-(bis(5-bromo-1H-indol-3-yl)methyl)phenyl)-1,3,4-thiadiazol-2-yl)-2,4-dinitrobenzenesulfonamide (8)

Orange solid, Yield: 83.0%; R_ƒ._ 0.50 (ethylecetate/hexane 3:7); m.p.: 315–314 °C; IR (KBr): 3242 cm^−1^ (2°amine N-H Str), 1610 cm^−1^ (Ar C=C), 1565 cm^−1^ (N-O str), 1358 cm^−1^ (N-S=O), 630 cm^−1^ (C-Br str); ^1^H NMR (500 MHz, DMSO-d_6_): *δ* 11.91 (s, 1H, NH), 11.62 (s, 1H, NH), 8.51 (s, 1H, NH), 7.85 (d, *J* = 6.8 Hz, 2H), 7.50 (d, *J* = 6.8 Hz, 2H), 7.38 (d, *J* = 8.3 Hz, 2H), 7.30 (d, J = 7.4 Hz, 2H), 6.92 (t, *J* = 7.5 Hz, 2H), 6.87 (s, 1H), 6.84 (d, *J* = 7.3 Hz, 1H), 6.72 (t, *J* = 7.5 Hz, 1H), 5.94 (s, 1H); ^13^C-NMR (125 MHz, DMSO-*d*6): *δ*174.1, 173.0, 152.0, 148.1, 140.5, 138.1, 135.5, 135.5, 130.5, 130.3, 129.5, 129.5, 129.1, 127.4, 127.4, 124.7, 124.7, 123.0, 123.0, 121.0, 121.0, 116.4, 116.4, 114.5, 113.2, 113.2, 112.1, 112.1, 54.6: HREI-MS: m/z Calcd for C_31_H_19_Br_2_N_7_O_6_S_2_ [M + 4]^+^ 810.9155, [M + 3]^+^ 809.9205, [M + 2]^+^ 808.9176, [M + 1]^+^ 806.9195 [M + ]^+^ 806.9198.

#### N-(5-(4-(bis(5-bromo-1H-indol-3-yl)methyl)phenyl)-1,3,4-thiadiazol-2-yl)-3-chloro-4-nitrobenzenesulfonamide (9)

Red solid. Yield: 79.0%; R_ƒ._ 0.58 (ethylecetate/hexane 3:7); m.p.: 318–319 °C; IR (KBr): 3210 cm^−1^ (2°amine N-H Str), 1617 cm^−1^ (Ar C=C), 1568 cm^−1^ (N-O str), 1365 cm^−1^ (N-S=O), 792 cm^−1^ (C-Cl str), 657 cm^−1^ (C-Br str); ^1^H NMR (500 MHz, DMSO-d6): *δ*11.46(s, 1H, NH), 9.47 (s, 1H, NH), 9.22 (s, 1H, NH), 7.83 (d, *J* = 8.4 Hz, 2H), 7.47 (d, *J* = 8.4 Hz, 2H), 7.36 (d, *J* = 8.1 Hz, 2H), 7.32 (d, *J* = 7.6 Hz, 2H), 7.21 (s, 1H), 6.96–6.92 (m, 3H), 6.85 (s, 2H), 6.76 (d, *J* = 8.3 Hz, 1H), 5.96 (s, 1H);^13^C-NMR (125 MHz, DMSO-*d*6): *δ*174.1, 173.0, 150.8, 147.2, 138.1, 135.5, 135.5, 130.5, 129.7, 129.6, 129.6, 129.5, 129.5, 127.5, 127.4, 127.4, 126.3, 125.6, 124.7, 124.7, 123.0, 123.0, 121.0, 121.0, 116.4, 116.4, 113.2, 113.2, 112.1, 112.1, 54.6: HREI-MS: m/z Calcd for C_31_H_19_Br_2_ClN_6_O_4_S_2_ [M + 4]^+^ 799.8902, [M + 3]^+^ 798.8969, [M + 2]^+^ 797.8932, [M + 1]^+^ 796.8986 [M]^+^ 795.8954.

#### N-(5-(4-(bis(5-bromo-1H-indol-3-yl)methyl)phenyl)-1,3,4-thiadiazol-2-yl)-3,4-dichlorobenzenesulfonamide (10)

Brown solid. Yield: 80.0%; R_ƒ._ 0.70 (ethylecetate/hexane 3:7); m.p.: 302–303 °C; IR (KBr): 3250 cm^−1^ (2°amine N-H Str), 1616 cm^−1^ (Ar C=C), 1355 cm^−1^ (N-S=O), 798 cm^−1^ (C-Cl str), 634 cm^−1^ (C-Br str); ^1^H NMR (500 MHz, DMSO-d_*6*_): *δ* 11.62(s, 1H, NH), 9.81 (s, 1H, NH), 9.70 (s, 1H, NH), 7.83 (d, *J* = 8.4 Hz, 2H),7.81 (d, *J* = 8.1 Hz, 2H), 7.48 (d, *J* = 8.1 Hz, 2H), 7.32–7.27 (m, 4H), 6.93 (t, *J* = 7.5 Hz, 2H), 6.87 (s, 2H), 6.57 (s, 2H), 6.24 (s, 1H), 5.94 (s, 1H); ^13^C-NMR (125 MHz, DMSO-*d6*): *δ*174.1, 173.0, 139.2, 138.1, 136.6, 135.5, 135.5, 133.7, 130.5, 130.5, 129.6, 129.6, 129.5, 129.5, 128.0, 127.4, 127.4, 126.8, 124.7, 124.7, 123.0, 123.0, 121.0, 121.0, 116.4, 116.4, 113.2, 113.2, 112.1, 112.1, 54.6: HREI-MS: m/z Calcd for C_31_H_19_Br_2_Cl_2_N_5_O_2_S_2_ [M + 6]^+^ 790.8642, [M + 5]^+^ 789.8695, [M + 4]^+^ 788.8654, [M + 3]^+^ 787.8719, [M + 2]^+^ 786.8694, [M + 1]^+^ 785.8744 [M + ]^+^ 784.8715.

#### N-(5-(4-(bis(5-bromo-1H-indol-3-yl)methyl)phenyl)-1,3,4-thiadiazol-2-yl)naphthalene-2-sulfonamide (11)

Orange solid. Yield: 80.0%; R_ƒ._ 0.61 (ethylecetate/hexane 2:8); m.p.: 313–314 °C; IR (KBr): 3240 cm^−1^ (2°amine N-H Str), 1640 cm^−1^ (Ar C=C), 1369 cm^−1^ (N-S=O), 670 cm^−1^ (C-Br str); ^1^H NMR (500 MHz, DMSO-d_*6*_): *δ*11.88(s, 1H, NH), 11.28 (s, 1H, NH), 10.48 (s, 1H, NH), 7.80 (t, *J* = 7.3 Hz, 3H), 7.52–7.45 (m, 6 H), 7.38 (d, *J* = 8.4 Hz, 2H), 7.31 (d, *J* = 7.5 Hz, 2H), 7.11 (t, J = 7.6 Hz, 2H), 6.93 (t, *J* = 7.2 Hz, 2H), 6.85 (s, 2H), 5.96 (s, 1H); ^13^C-NMR (125 MHz, DMSO-*d6*): *δ*174.1, 173.0, 138.1, 137.0, 136.7, 135.5, 135.5, 134.1, 130.5, 129.6, 129.6, 129.5, 129.5, 129.4, 128.1, 128.1, 127.4, 127.4, 126.2, 126.2, 126.0, 124.7, 124.7, 123.4, 123.0, 123.0, 121.0, 121.0, 116.4, 116.4, 113.2, 113.2, 112.1, 112.1, 54.6: HREI-MS: m/z Calcd for C_35_H_23_Br_2_N_5_O_2_S_2_ [M + 4]^+^ 770.9606, [M + 3]^+^ 769.9662, [M + 2]^+^ 767.9683, [M + 1]^+^ 785.8744 [M + ]^+^ 766.952.

#### N-(5-(4-(bis(5-bromo-1H-indol-3-yl)methyl)phenyl)-1,3,4-thiadiazol-2-yl)-2-chloro-5-methylbenzenesulfonamide (12)

Yellow solid. Yield: 83.0%; R_ƒ._ 0.68 (ethylecetate/hexane 3:7); m.p.: 301–302 °C; IR (KBr): 3236 cm^−1^ (2°amine N-H Str), 1632 cm^−1^ (Ar C=C), 1349 cm^−1^ (N-S=O), 770 cm^−1^ (C-Cl str), 639 cm^−1^ (C-Br str); ^1^H NMR (500 MHz, DMSO-d_*6*_): *δ*11.72(s, 1H, NH), 11.89 (s, 1H, NH), 9.61 (s, 1H, NH), 7.80 (d, *J* = 8.3 Hz, 2H), 7.51 (d, *J* = 8.3 Hz, 2H), 7.39 (d, *J* = 8.4 Hz, 2H), 7.33 (d, *J* = 7.6 Hz, 2H), 7.18–7.11 (m, 3H), 6.91 (t, *J* = 7.2 Hz, 2H), 6.85(s, 2H), 5.96 (s, 1H), 1.92 (s, 3H, CH_3_); ^13^C-NMR (125 MHz, DMSO-*d6*): *δ*174.1, 173.0, 139.6, 138.1, 136.8, 135.5, 135.5, 133.6, 130.5, 129.6, 129.6, 129.5, 129.5, 129.0, 128.5, 128.1, 127.4, 127.4, 124.7, 124.7, 123.0, 123.0, 121.0, 121.0, 116.4, 116.4, 113.2, 113.2, 112.1, 112.1, 54.6, 21.3: HREI-MS: m/z Calcd for C_32_H_22_Br_2_ClN_5_O_2_S_2_ [M + 4]^+^ 768.9219, [M + 3]^+^ 767.9272, [M + 2]^+^ 766.9240, [M + 1]^+^ 765.9297 [M + ]^+^ 764.918.

#### N-(5-(4-(bis(5-bromo-1H-indol-3-yl)methyl)phenyl)-1,3,4-thiadiazol-2-yl)-5 methyl-2-nitrobenzenesulfonamide (13)

Orange solid. Yield: 78.0%; R_ƒ._ 0.59 (ethylecetate/hexane 3:7); m.p.: 312–313 °C; IR (KBr): 3237 cm^−1^ (2°amine N-H Str), 1623 cm^−1^ (Ar C=C), 1563 cm^−1^ (N-O str), 1345 cm^−1^ (N-S=O), 687 cm^−1^ (C-Br str); ^1^H NMR (500 MHz, DMSO-d_*6*_): *δ*12.02(s, 1H, NH), 11.12 (s, 1H, NH), 10.98 (s, 1H, NH), 7.82 (d, *J* = 7.8 Hz, 2H), 7.74 (t, *J* = 7.6 Hz, 1H), 7.50 (d, *J* = 8.3 Hz, 2H), 7.38 (d, *J* = 8.6 Hz, 2H), 7.32 (d, *J* = 7.7 Hz, 2H), 7.11 (t, *J* = 7.4 Hz, 2H), 6.94 (t, *J* = 7.4 Hz, 2H), 6.87 (s, 2H), 5.94 (s, 1H), 1.91 (s, 3H, CH_3_);^13^C-NMR (125 MHz, DMSO-*d6*): *δ*174.1, 173.0, 144.8, 144.2, 138.1, 135.5, 135.5, 134.3, 133.1, 130.5, 129.6, 129.6, 129.5, 129.5, 127.6, 127.4, 127.4, 124.7, 124.7, 124.1, 123.0, 123.0, 121.0, 121.0, 116.4, 116.4, 113.2, 113.2, 112.1, 112.1, 54.6, 21.3: HREI-MS: m/z Calcd for C_32_H_22_Br_2_N_6_O_4_S_2_ [M + 4]^+^ 768.9219, [M + 3]^+^ 779.9461, [M + 2]^+^ 778.9514, [M + 1]^+^ 776.9527 [M + ]^+^ 775.9502.

#### N-(5-(4-(bis(5-bromo-1H-indol-3-yl)methyl)phenyl)-1,3,4-thiadiazol-2-yl)-2-bromo-5-methoxybenzenesulfonamide (14)

Yellow solid. Yield: 87.0%; R_ƒ._ 0.71 (ethylecetate/hexane 3:7); m.p.:309–310 °C; IR (KBr): 3190 cm^−1^ (2°amine N-H Str), 1609 cm^−1^ (Ar C=C), 1341 cm^−1^ (N-S=O), 716 cm^−1^ (C-Br str); ^1^H NMR (500 MHz, DMSO-d_*6*_): *δ* 11.95 (s, 1H, NH), 10.68 (s, 1H, NH), 8.61(s, 1H, NH), 7.82 (d, *J* = 7.3 Hz, 2H), 7.50 (d, *J* = 8.2 Hz, 2H), 7.38 (d, *J* = 8.6 Hz, 2H), 7.33 (d, J = 7.7 Hz, 2H), 7.14–7.09 (m, 3H), 6.94–6.90 (m, 2H), 6.86 (s, 2H), 6.85 (d, J = 7.4 Hz, 1H), 5.96 (s, 1H), 3.74 (s, 3H, CH_3_); ^13^C-NMR (125 MHz, DMSO-*d6*): *δ*174.1, 173.0, 158.3, 143.6, 138.1, 135.5, 135.5, 132.9, 130.5, 129.6, 129.6, 129.5, 129.5, 127.4, 127.4, 124.7, 124.7, 123.0, 123.0, 121.5, 121.0, 121.0, 116.4, 116.4, 113.4, 113.2, 113.2, 112.4, 112.1, 112.1, 55.8, 54.6: HREI-MS: m/z Calcd for C_32_H_22_Br_3_N_5_O_3_S_2_ [M + 6]^+^ 830.8641, [M + 5]^+^ 829.8695, [M + 4]^+^ 828.8662, [M + 3]^+^ 827.8709, [M + 2]^+^ 826.8679, [M + 1]^+^ 825.8732 [M + ]^+^ 824.8714.

#### N-(5-(4-(bis(5-bromo-1H-indol-3-yl)methyl)phenyl)-1,3,4-thiadiazol-2-yl)-5-methoxy-2-nitrobenzenesulfonamide (15)

Brown solid. Yield: 81.0%; R_ƒ._ 0.58 (ethylecetate/hexane 3:7); m.p.: 308–309 °C; IR (KBr): 3234 cm^−1^ (2°amine N-H Str), 1625 cm^−1^ (Ar C=C), 1554 cm^−1^ (N-O str), 1359 cm^−1^ (N-S=O), 1129 cm^−1^ (C-O str), 682 cm^−1^ (C-Br str); ^1^H NMR (500 MHz, DMSO-d_*6*_): *δ*11.62(s, 1H, NH), 11.47 (s, 1H, NH), 8.38 (s, 1H, NH), 7.80 (d, *J* = 8.4 Hz, 2H), 7.64 (d, *J* = 7.8 Hz, 2H), 7.49 (d, *J* = 8.1 Hz, 2H), 7.38 (d, *J* = 8.3 Hz, 2H), 7.31 (d, *J* = 7.7 Hz, 2H), 7.04 (d, *J* = 8.6 Hz, 2H), 6.90 (t, *J* = 7.2 Hz, 2H), 6.86 (s, 2H), 5.96 (s, 1H), 3.82 (s, 3H, OCH_3_);^13^C-NMR (125 MHz, DMSO-*d6*): *δ*174.1, 173.0, 165.4, 139.5, 138.1, 135.5, 135.5, 135.4, 130.5, 129.6, 129.6, 129.5, 129.5, 127.4, 127.4, 125.2, 124.7, 124.7, 123.0, 123.0, 121.0, 121.0, 118.4, 116.4, 116.4, 113.2, 113.2, 112.1, 112.1, 112.1, 55.8, 54.6: HREI-MS: m/z Calcd for C_32_H_22_Br_2_N_6_O_5_S_2_ [M + 4]^+^ 795.9402, [M + 3]^+^ 794.9464, [M + 2]^+^ 793.9423, [M + 1]^+^ 792.9482 [M + ]^+^ 791.932.

#### N-(5-(4-(bis(5-bromo-1H-indol-3-yl)methyl)phenyl)-1,3,4-thiadiazol-2-yl)-2-methylbenzenesulfonamide (16)

Light brown. Yield: 82.0%; R_ƒ._ 0.75 (ethylecetate/hexane 3:7); m.p.: 291–292 °C; IR (KBr): 3232 cm^−1^ (2°amine N-H Str), 1620 cm^−1^ (Ar C=C), 1354 cm^−1^ (N-S=O), 722 cm^−1^ (C-Br str); ^1^H NMR (500 MHz, DMSO-d_*6*_): *δ*11.72(s, 1H, NH), 10.96 (s, 1H, NH), 8.72 (s, 1H, NH), 7.81 (t, *J* = 7.4 Hz, 2H), 7.49 (d, *J* = 7.7 Hz, 2H), 7.38 (d, *J* = 8.3 Hz, 2H), 7.31 (d, *J* = 7.6 Hz, 2H), 7.30–7.21 (m, 3H), 6.94 (t, *J* = 7.3 Hz, 2H), 6.86 (s, 2H), 5.96 (s, 1H), 2.40 (s, 3H, CH_3_); ^13^C-NMR (125 MHz, DMSO-*d6*): *δ*174.1, 173.0, 138.9, 138.1, 136.6, 131.8, 131.5, 130.5, 129.7, 129.6, 129.6, 129.5, 129.5, 127.4, 127.4, 124.7, 124.7, 123.0, 123.0, 121.0, 121.0, 120.8, 116.4, 116.4, 113.2, 113.2, 112.1, 112.1, 54.6, 22.0: HREI-MS: m/z Calcd for C_32_H_23_Br_2_N_5_O_2_S_2_ [M + 4]^+^ 734.9606, [M + 3]^+^ 733.9662, [M + 2]^+^ 732.9624, [M + 1]^+^ 731.9682 [M + ]^+^ 730.9652.

#### N-(5-(4-(bis(5-bromo-1H-indol-3-yl)methyl)phenyl)-1,3,4-thiadiazol-2-yl)-3-methylbenzenesulfonamide (17)

Yellow. Yield: 82.0%; R_ƒ._ 0.74 (ethylecetate/hexane 3:7); m.p.: 289–290 °C; IR (KBr): 3236 cm^−1^ (2°amine N-H Str), 1629 cm^−1^ (Ar C=C), 1361 cm^−1^ (N-S=O), 730 cm^−1^ (C-Br str); ^1^H NMR (500 MHz, DMSO-d_*6*_): *δ* 12.52 (s, 1H, NH), 11.74 (s, 1H, NH), 8.42 (s, 1H, NH),7.80 (d, *J* = 7.7 Hz, 2H), 7.52 (s, 1H), 7.49 (d, *J* = 8.4 Hz, 2H), 7.39 (d, *J* = 8.3 Hz, 2H), 7.31 (d, J = 7.6 Hz, 2H), 7.22 (d, *J* = 8.3 Hz, 2H), 6.91 (t, *J* = 7.2 Hz, 2H), 6.86 (s, 2H), 5.95 (s, 1H), 2.40 (s, 3H, CH_3_); ^13^C-NMR (125 MHz, DMSO-*d6*): *δ*174.1, 173.0, 139.6, 138.1, 138.7, 135.5, 135.5, 132.2, 130.5, 129.6, 129.6, 129.5, 129.5, 128.9, 127.4, 127.4, 126.7, 124.7, 124.7, 124.3, 123.0, 123.0, 121.0, 121.0, 116.4, 116.4, 113.2, 113.2, 112.1, 112.1, 54.6, 21.3: HREI-MS: m/z Calcd for C_32_H_23_Br_2_N_5_O_2_S_2_ [M + 4]^+^ 734.9609, [M + 3]^+^ 733.9659, [M + 2]^+^ 732.9622, [M + 1]^+^ 731.9679 [M + ]^+^ 730.9647.

#### N-(5-(4-(bis(5-bromo-1H-indol-3-yl)methyl)phenyl)-1,3,4-thiadiazol-2-yl)-4-methylbenzenesulfonamide (18)

Brown. Yield: 75.0%; R_ƒ._ 0.72 (ethylecetate/hexane 3:7); m.p.: 285–286 °C; IR (KBr): 3220 cm^−1^ (2°amine N-H Str), 1631 cm^−1^ (Ar C=C), 1359 cm^−1^ (N-S=O), 726 cm^−1^ (C-Br str); ^1^H NMR (500 MHz, DMSO-*d*_*6*_): *δ* 12.08 (s, 1H, NH), 11.74 (s, 1H, NH), 8.37 (s, 1H, NH),7.83 (d, *J* = 8.3 Hz, 2H), 7.60 (d, *J* = 7.3 Hz, 2H), 7.48 (d, *J* = 7.6 Hz, 2H), 7.39 (d, *J* = 7.8 Hz, 2H), 7.30 (d, *J* = 7.4 Hz, 2H), 7.24 (d, *J* = 7.3 Hz, 2H), 6.93 (t, *J* = 7.3 Hz, 2H), 6.85 (s, 2H), 5.94 (s, 1H), 2.37 (s, 3H, CH_3_);^13^C-NMR (125 MHz, DMSO-*d6*): *δ*174.1, 173.0, 138.1, 137.6, 136.7, 135.5, 135.5, 130.5, 129.6, 129.6, 129.5, 129.5, 129.3, 129.3, 128.3, 128.3, 127.4, 127.4, 124.7, 124.7, 123.0, 123.0, 121.0, 121.0, 116.4, 116.4, 113.2, 113.2, 112.1, 112.1, 54.6, 21.3: HREI-MS: m/z Calcd for C_32_H_23_Br_2_N_5_O_2_S_2_ [M + 4]^+^ 734.9602, [M + 3]^+^ 733.9656, [M + 2]^+^ 732.9618, [M + 1]^+^ 731.9675 [M + ]^+^ 730.9649.

### Urease inhibition assay

Urease is an enzyme (jack bean urease) that catalyzes the hydrolysis of urea into carbon dioxide and ammonia. The production of ammonia was measured by the indophenol method and used to determine the urease inhibitory activity^57,58^. The percentage remaining activity was calculated from the formula % Remaining Activity = [(ODtest)/(ODcontrol) × 100]. Thiourea was used as a standard inhibitor. To calculate IC_50_ values, different concentrations of synthesized compounds and standards were assayed at the same reaction conditions.

### Docking studies

The binding modes between selected *bis*-indole bearing thiadiazol derivatives and the active residues of urease have been investigated using Autodock package^59^. The staring geometries of urease and the original docked acetohydroxamic acid were download from the RCSB data bank web site (PDB code 1FWE)^60^. Water molecules were removed; polar hydrogen atoms and Kollman charge were added to the extracted receptor using the automated tool in AutoDock Tools 4.2. The active site is identified based on co-crystallized receptor-ligand complex structure of urease. The re-docking of the original ligand acetohydroxamic acid into the active site is well reproduced with a RMSD value less than 0.717 Å. Molecular geometries of selected diindolylmethane bearing thiadiazol derivatives were minimized at Merck molecular force field 94 (MMFF94) level44. The optimized structures were saved as PDB files. Nonpolar hydrogens were merged and rotatable bonds were defined for each docked ligand. Docking studies were performed by Lamarckian genetic algorithm, with 500 as total number of run for binding sites for original ligand the synthesized derivatives. In each respective run, a population of 150 individuals with 27000 generations and 250000 energy evaluations were employed. Operator weights for crossover, mutation, and elitism were set to 0.8, 0.02, and 1, respectively. The docking calculations have been carried out using an Intel Core i5–3770 CPU 3.40 GHz workstation.

## Supplementary information

Supplementary information.
