# Supplementary material for: Synthesis of diindolylmethane (DIM) bearing thiadiazole derivatives as a potent urease inhibitor
Source: Sci Rep. 2020 May 14;10:7969. doi: 10.1038/s41598-020-64729-3 (PMC7224224; doi:10.1038/s41598-020-64729-3)
Supplement: Supplementary file 1 — Supplementary information. [file 41598_2020_64729_MOESM1_ESM.pdf]

Synthesis of diindolylmethane (DIM) bearing thiadiazole derivatives as a potent urease inhibitor

Muhammad Taha*[a], Fazal Rahim[b], Aftab Ahmad Khan[b], El Hassane Anoure[c], Naveed Ahmed[d], Syed Adnan Ali Shah[e,f], Mohamed Ibrahim[a], Zainul Amiruddin Zakaria*[g,h]

[a]Department of clinical pharmacy, Institute for Research and Medical Consultations (IRMC), Imam Abdulrahman Bin Faisal University, P.O. Box 31441, Dammam, Saudi Arabia.

[b]*Department of Chemistry, Hazara University, Mansehra-21300, Khyber Pakhtunkhwa, Pakistan*

[c]*Department of Chemistry, College of Science and Humanities, Prince Sattam bin Abdulaziz University, P.O. Box 83, Al Kharj 11942, Saudi Arabia*

[d]*Department of Pharmacy, Quaid-i-Azam University, Islamabad 45320, Pakistan*

[e]*Atta-ur-Rahman Institute for Natural Product Discovery, Universiti Teknologi MARA (UiTM), Puncak Alam Campus, 42300 Bandar Puncak Alam, Selangor D. E. Malaysia.*

[f]*Faculty of Pharmacy, Universiti Teknologi MARA (UiTM), Puncak Alam Campus, 42300 Bandar Puncak Alam, Selangor Darul Ehsan, Malaysia*

[g]*Department of Biomedical Science, Faculty of Medicine and Health Sciences, Universiti Putra Malaysia, 43400 Serdang, Selangor, Malaysia*

[h]*Halal Institute Research Institute, Universiti Putra Malaysia, 43400 Serdang, Selangor, Malaysia*

* Corresponding authors:
Dr Muhammad taha E-mail: taha_hej@yahoo.com and mtaha@iau.edu.sa, (MT)
*Correspondence 2: zaz@upm.edu.my (Tel.: +603-8947 2111; Fax: +603-89436178)

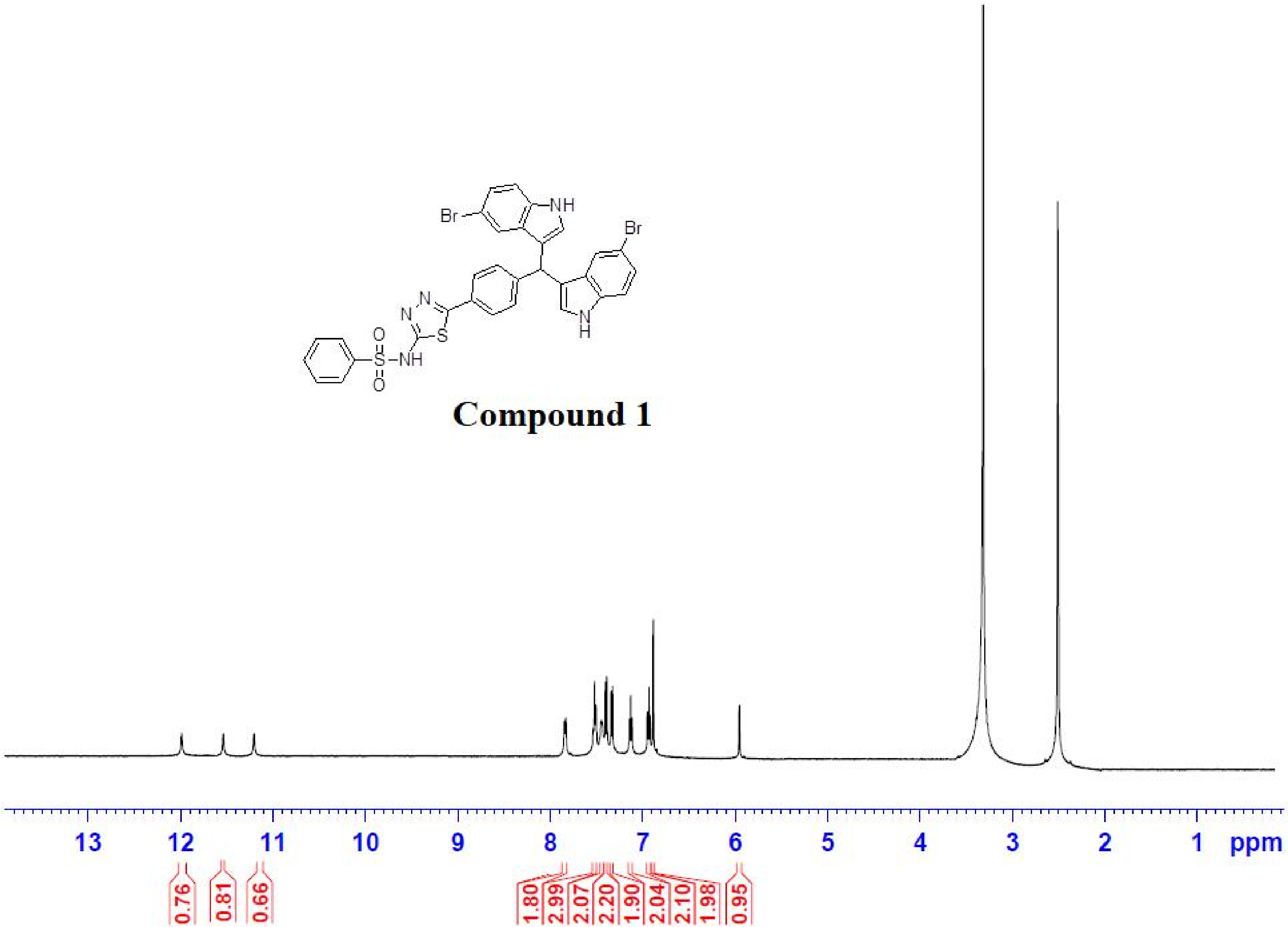
Br
NH
Br
N
N
S
O
S
NH
O
H
N
13 12 11 10 9 8 7 6 5 4 3 2 1 ppm
0.76
0.81
0.66
1.80
2.99
2.07
2.20
1.90
2.04
2.10
1.98
0.95

Compound 1

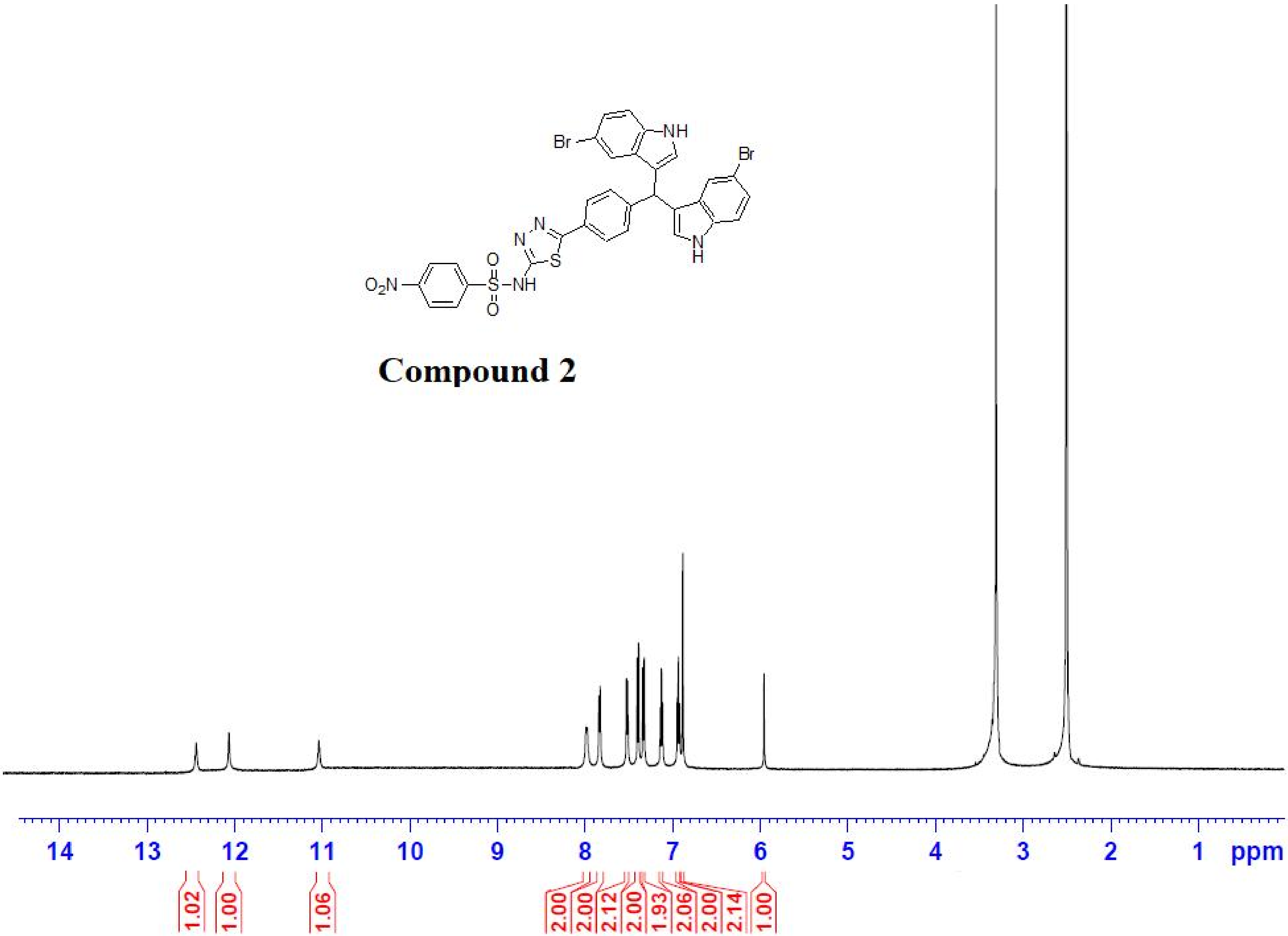
Br
NH
Br
N
N
N
H
O
O$_2$N
S
NH
O
S
Compound 2
14
13
12
11
10
9
8
7
6
5
4
3
2
1
ppm
1.02
1.00
1.06
2.00
2.00
2.12
2.00
1.93
2.06
2.00
2.14
1.00

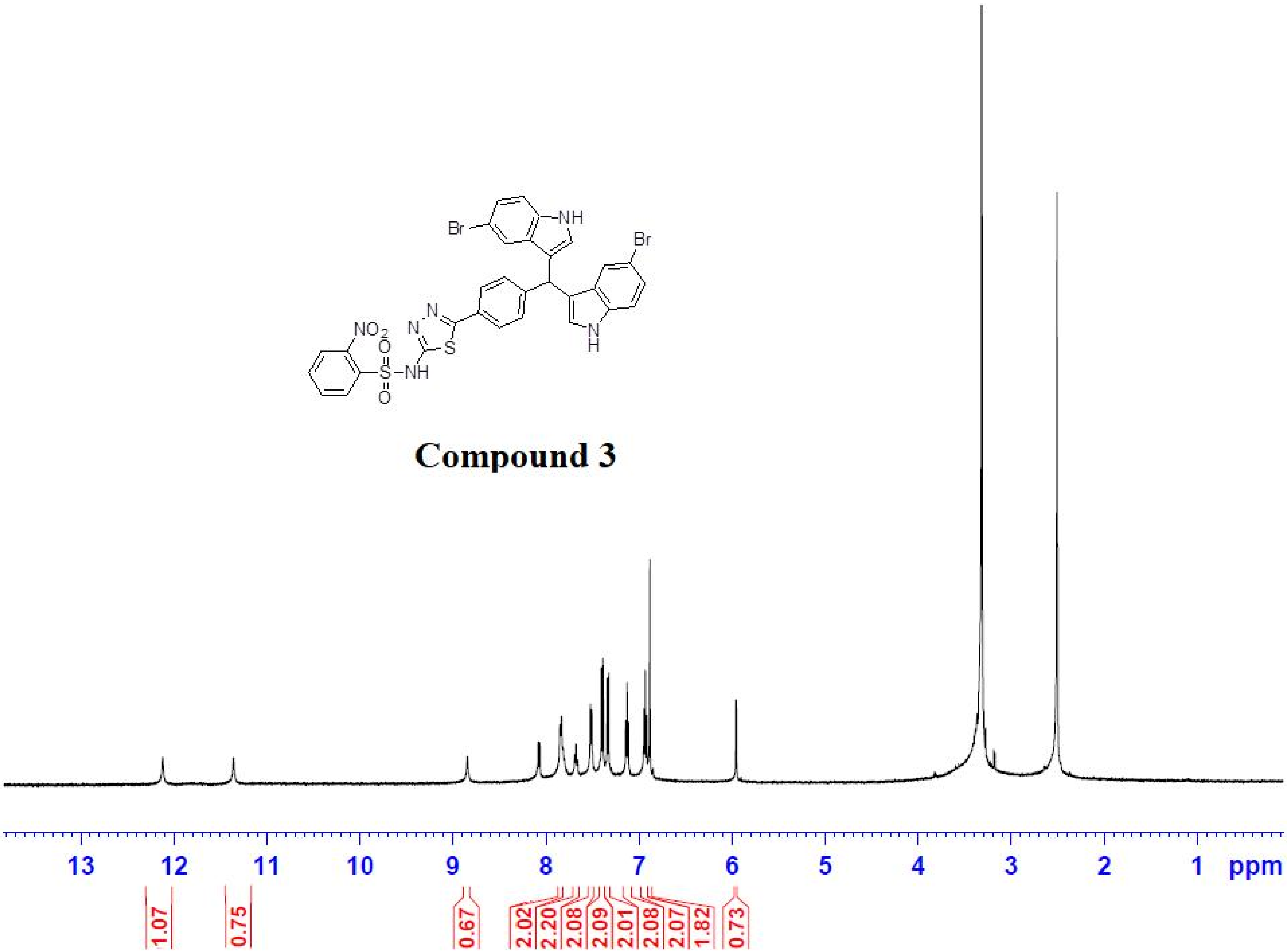
Br
NH
Br
N
N
NO2
S
O
S
NH
O
N
H
Compound 3
13
12
11
10
9
8
7
6
5
4
3
2
1
ppm
1.07
0.75
0.67
2.02
2.20
2.08
2.09
2.01
2.08
2.07
1.82
0.73

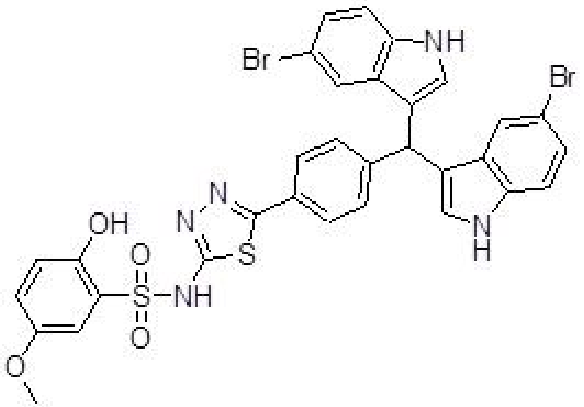

Br
NH
Br
N
N
OH
O
S
N
S
NH
O
O
H

Compound 4

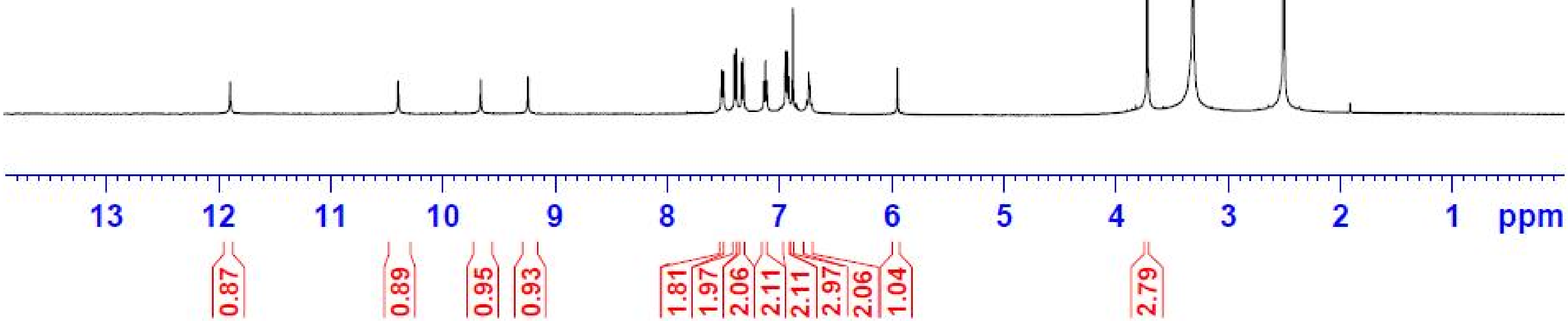

13 12 11 10 9 8 7 6 5 4 3 2 1 ppm
0.87
0.89
0.95
0.93
1.81
1.97
2.06
2.11
2.11
2.97
2.06
1.04
2.79

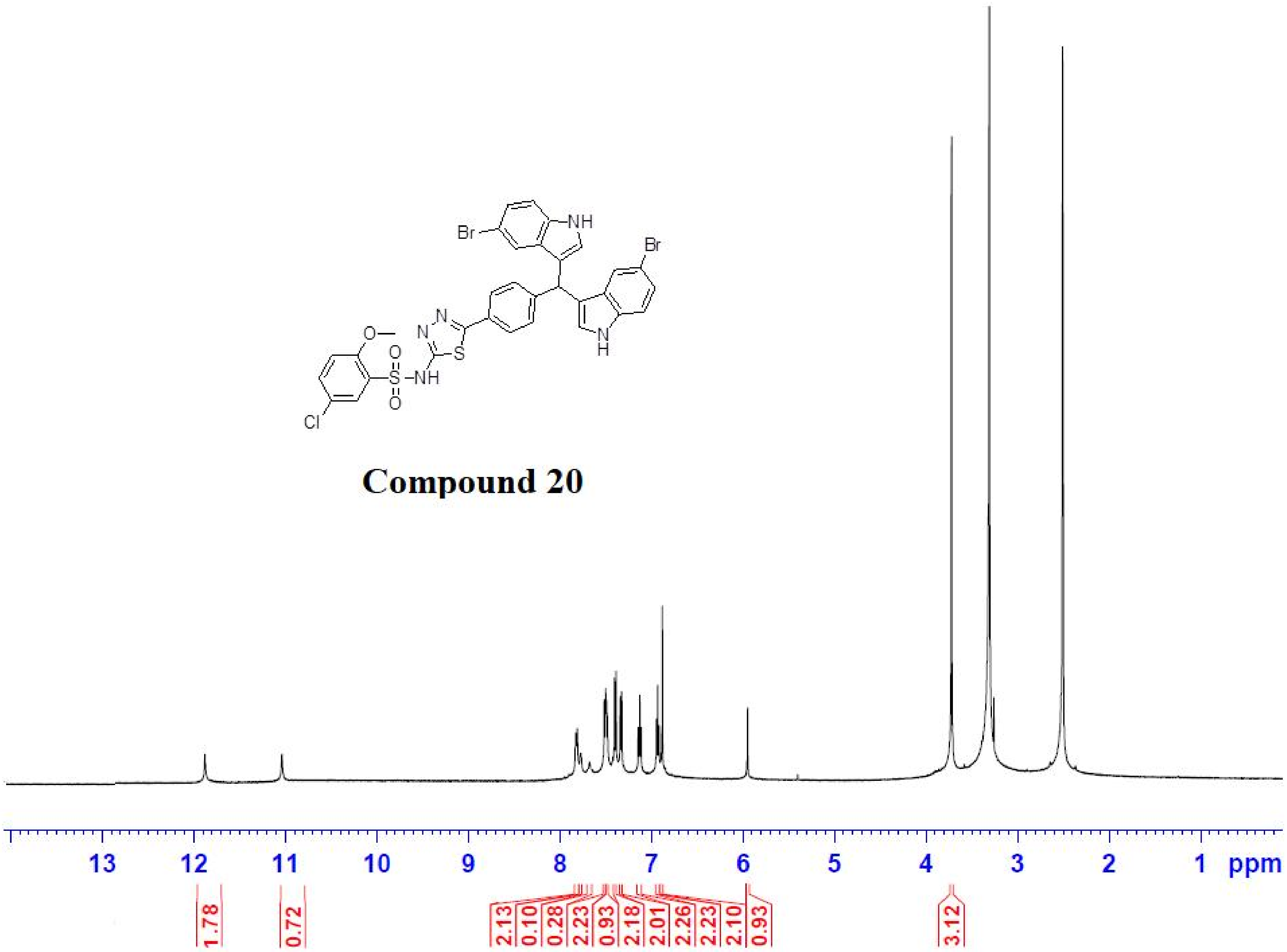
Br
NH
Br
N
N
N
H
O
O
S
NH
S
O
Cl
Compound 20
13
12
11
10
9
8
7
6
5
4
3
2
1
ppm
1.78
0.72
2.13
0.10
0.28
2.23
0.93
2.18
2.01
2.26
2.23
2.10
0.93
3.12

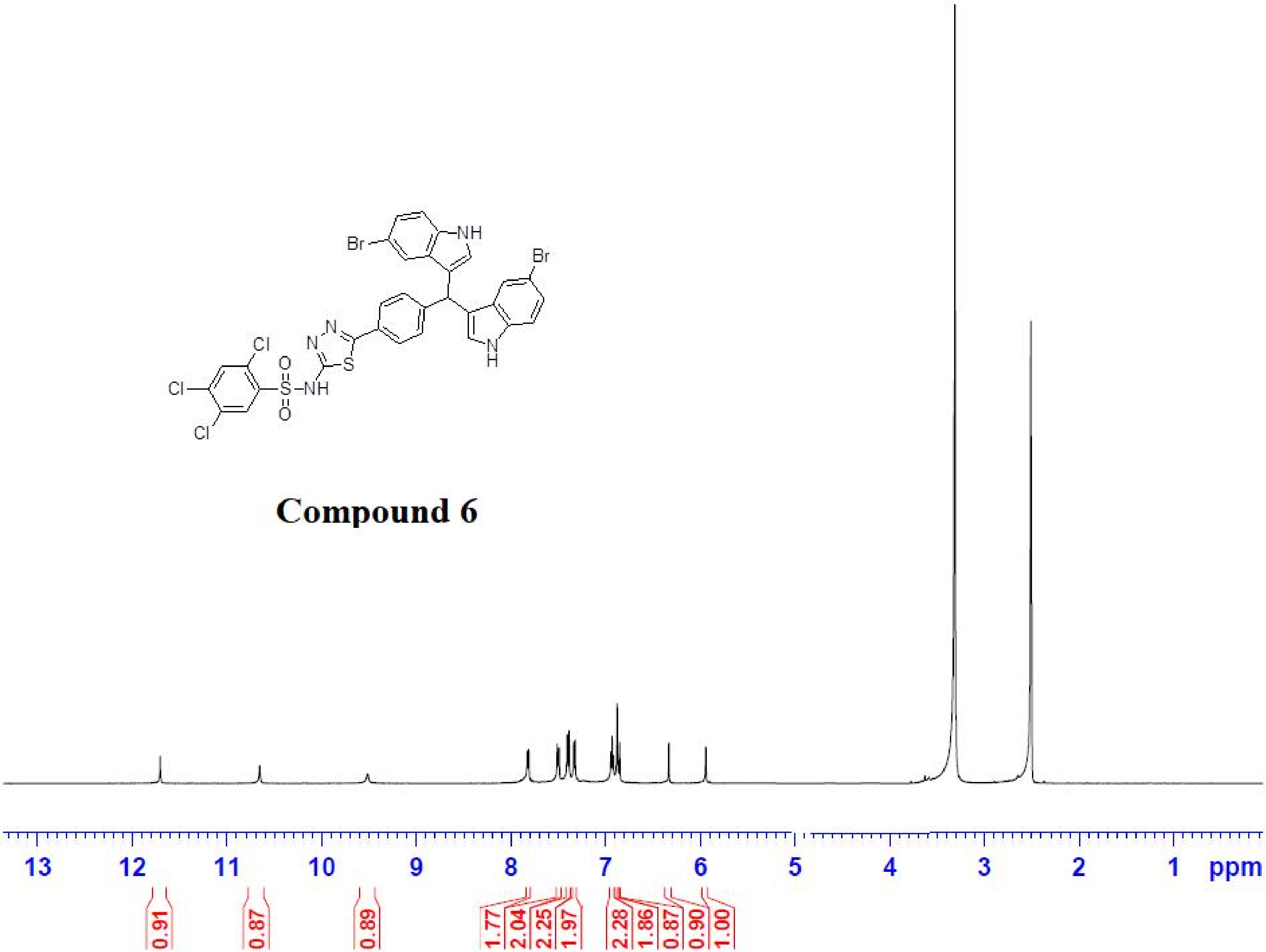
Br
NH
Br
N
N
Cl
S
O
Cl
S
NH
O
Cl
N
H
Compound 6
13
12
11
10
9
8
7
6
5
4
3
2
1
ppm
0.91
0.87
0.89
1.77
2.04
2.25
1.97
2.28
1.86
0.87
0.90
1.00

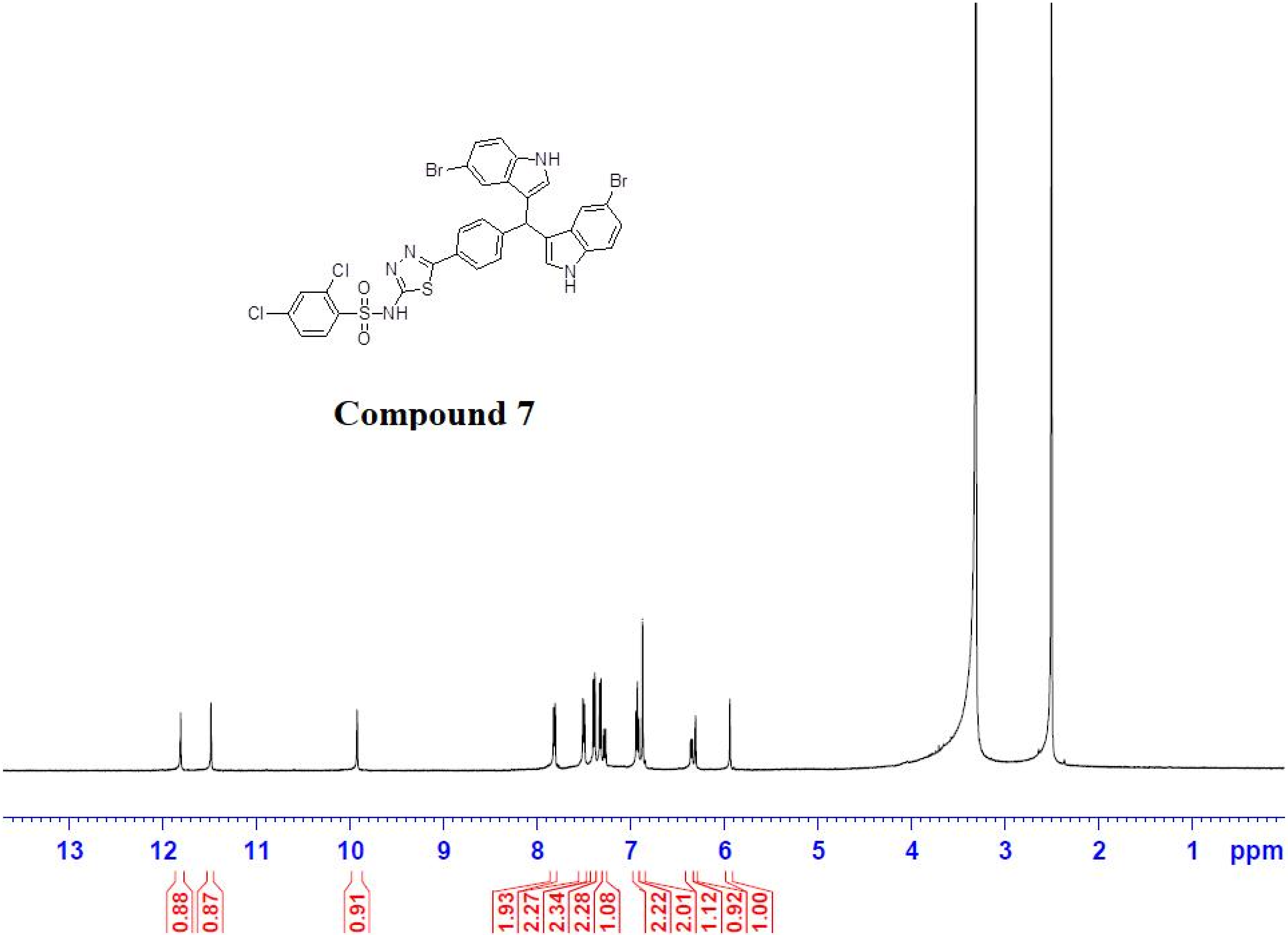

Br
NH
Br
N
N
Cl
N
S
O
Cl
S
NH
O
H
13 12 11 10 9 8 7 6 5 4 3 2 1 ppm
0.88 0.87 0.91 1.93 2.27 2.34 2.28 1.08 2.22 2.01 1.12 0.92 1.00

Compound 7

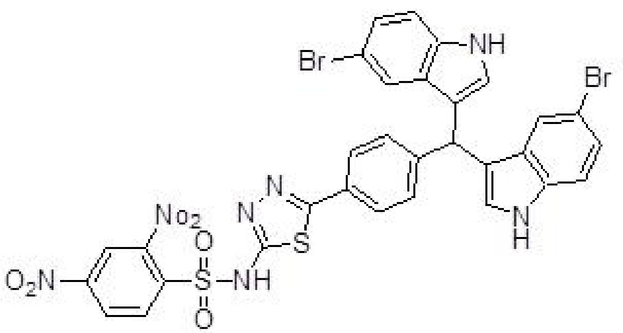
Br
NH
Br
N
N
No2
O
S
H
O2N
S
NH
O
N
H

Compound 8

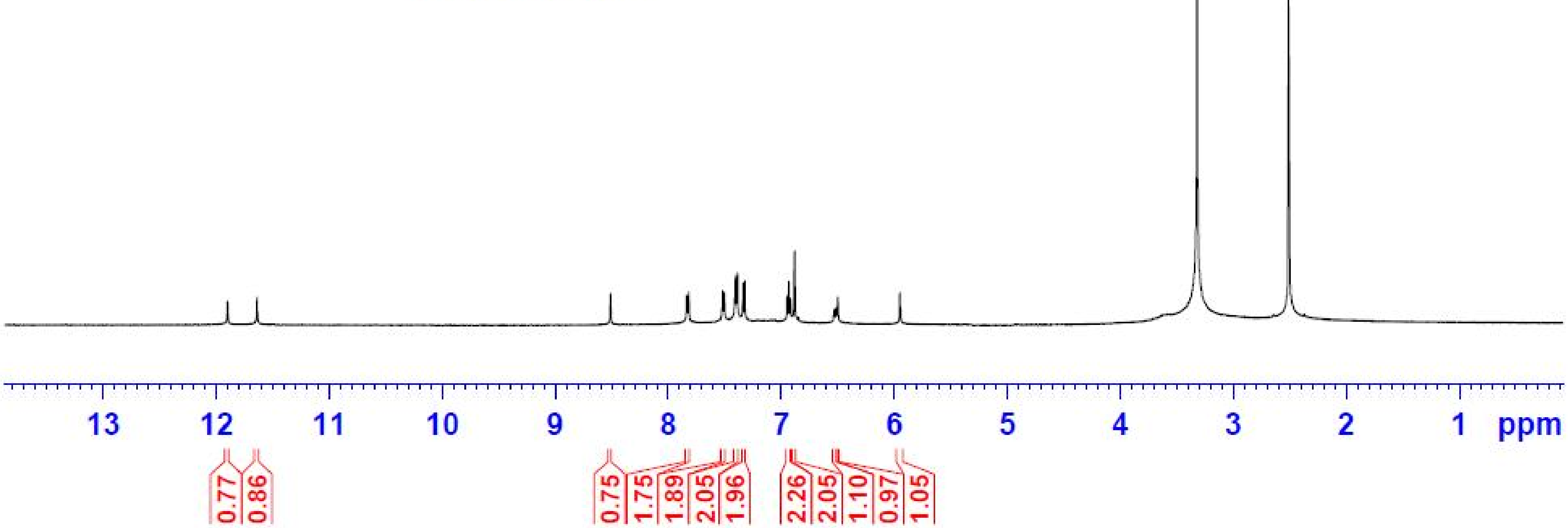
13
12
11
10
9
8
7
6
5
4
3
2
1
ppm
0.77
0.86
0.75
1.75
1.89
2.05
1.96
2.26
2.05
1.10
0.97
1.05

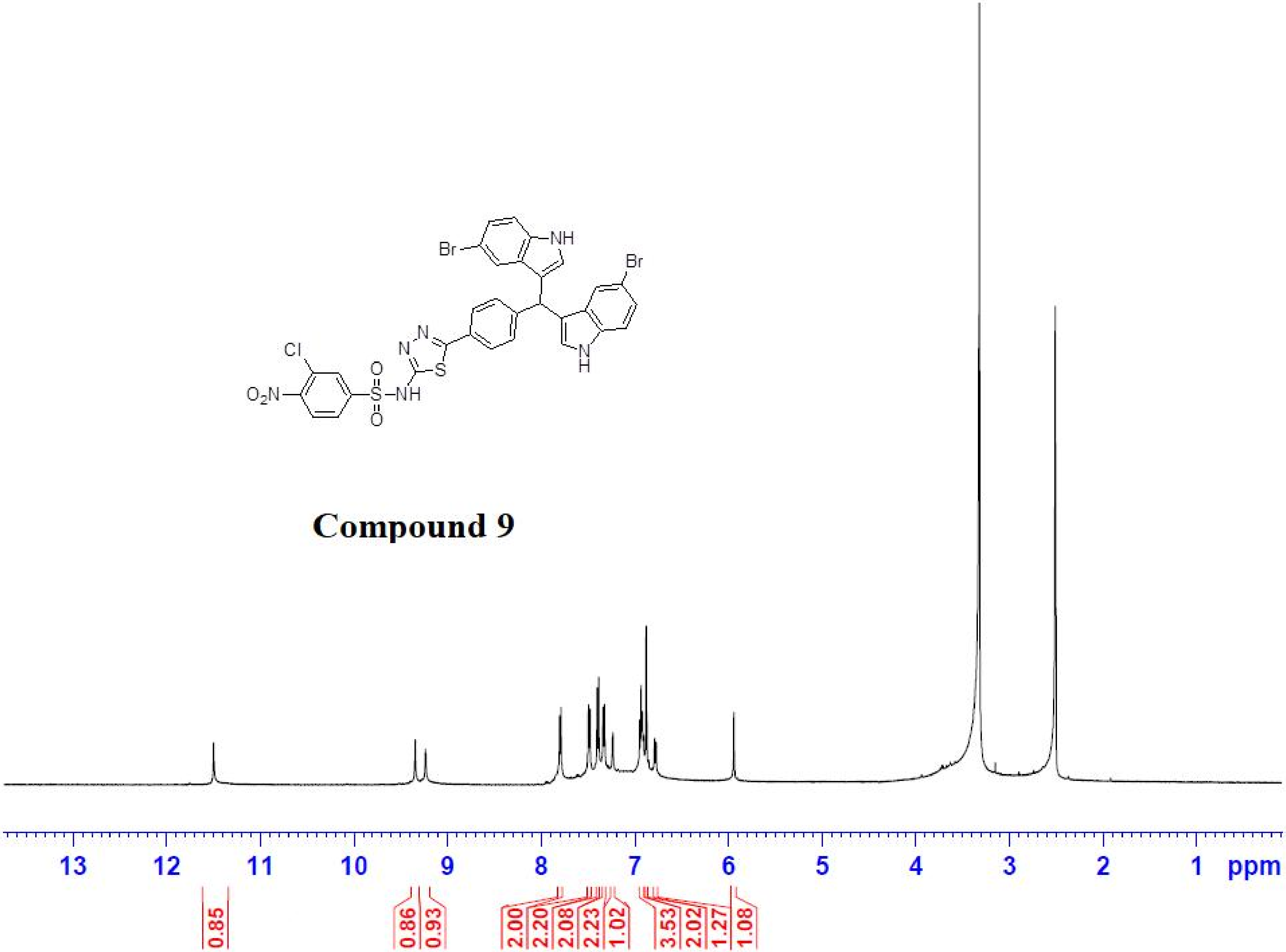
Compound 9
Br
NH
Br
N
H
N
N
Cl
O
O$_2$N
S
NH
S
O
13
12
11
10
9
8
7
6
5
4
3
2
1
ppm
0.85
0.86
0.93
2.00
2.20
2.08
2.23
1.02
3.53
2.02
1.27
1.08

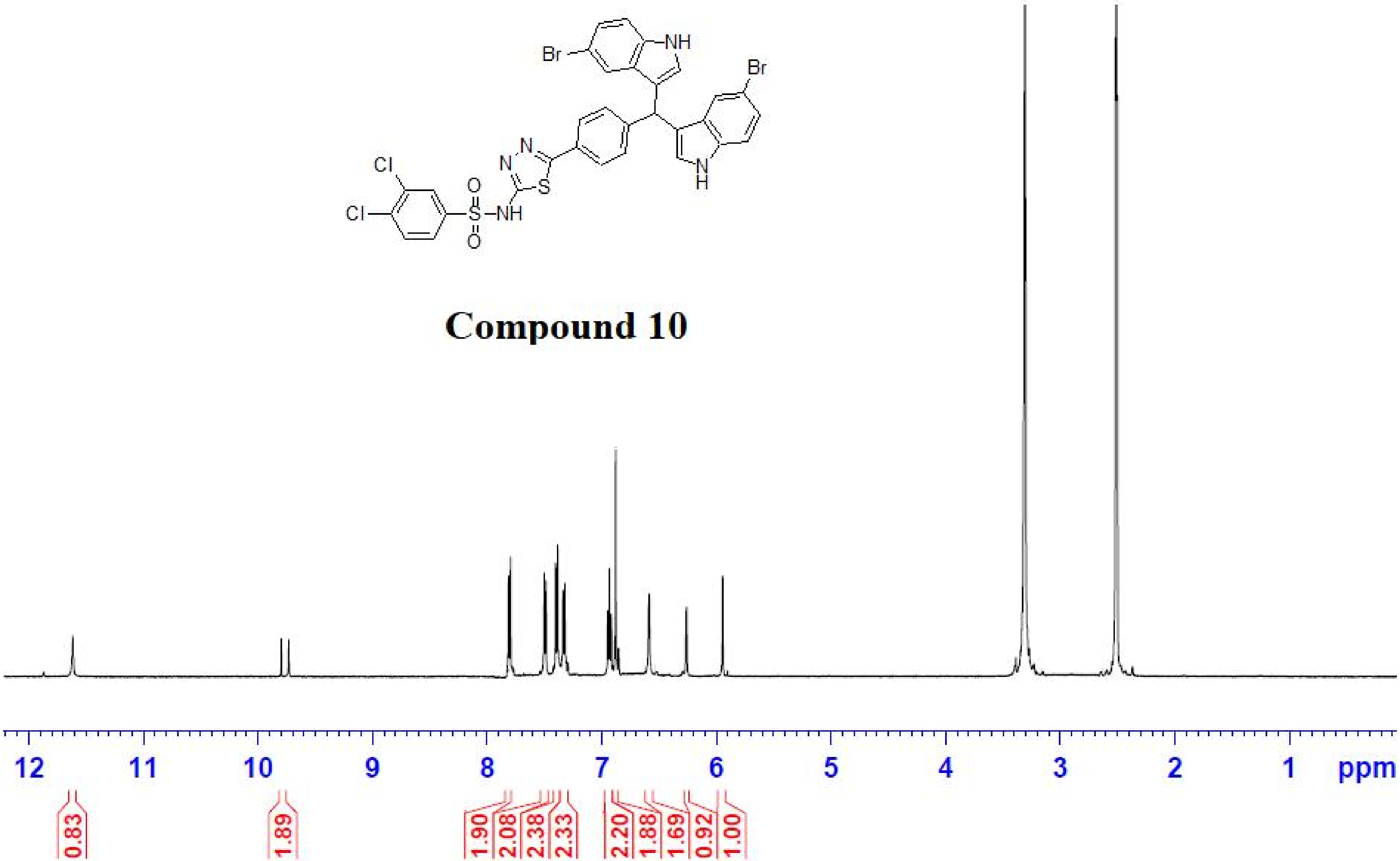
Br
NH
Br
N
N
Cl
O
Cl
S
NH
O
N
H
12
11
10
9
8
7
6
5
4
3
2
1
ppm
0.83
1.89
1.90
2.08
2.38
2.33
2.20
1.88
1.69
0.92
1.00

Compound 10

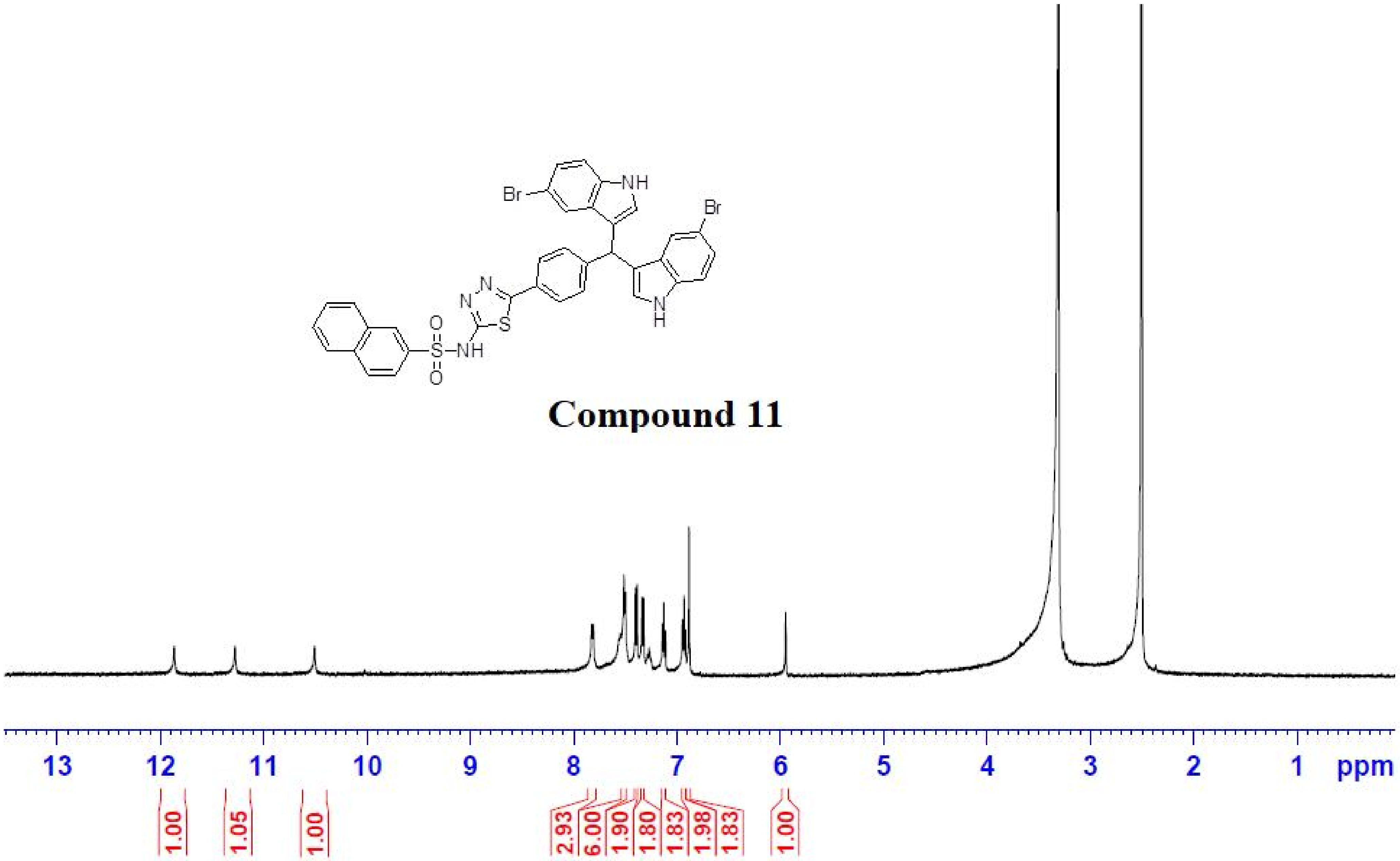
Br
NH
Br
N
N
S
O
S
NH
O
N
H
Compound 11
13
12
11
10
9
8
7
6
5
4
3
2
1
ppm
1.00
1.05
1.00
2.93
6.00
1.90
1.80
1.83
1.98
1.83
1.00

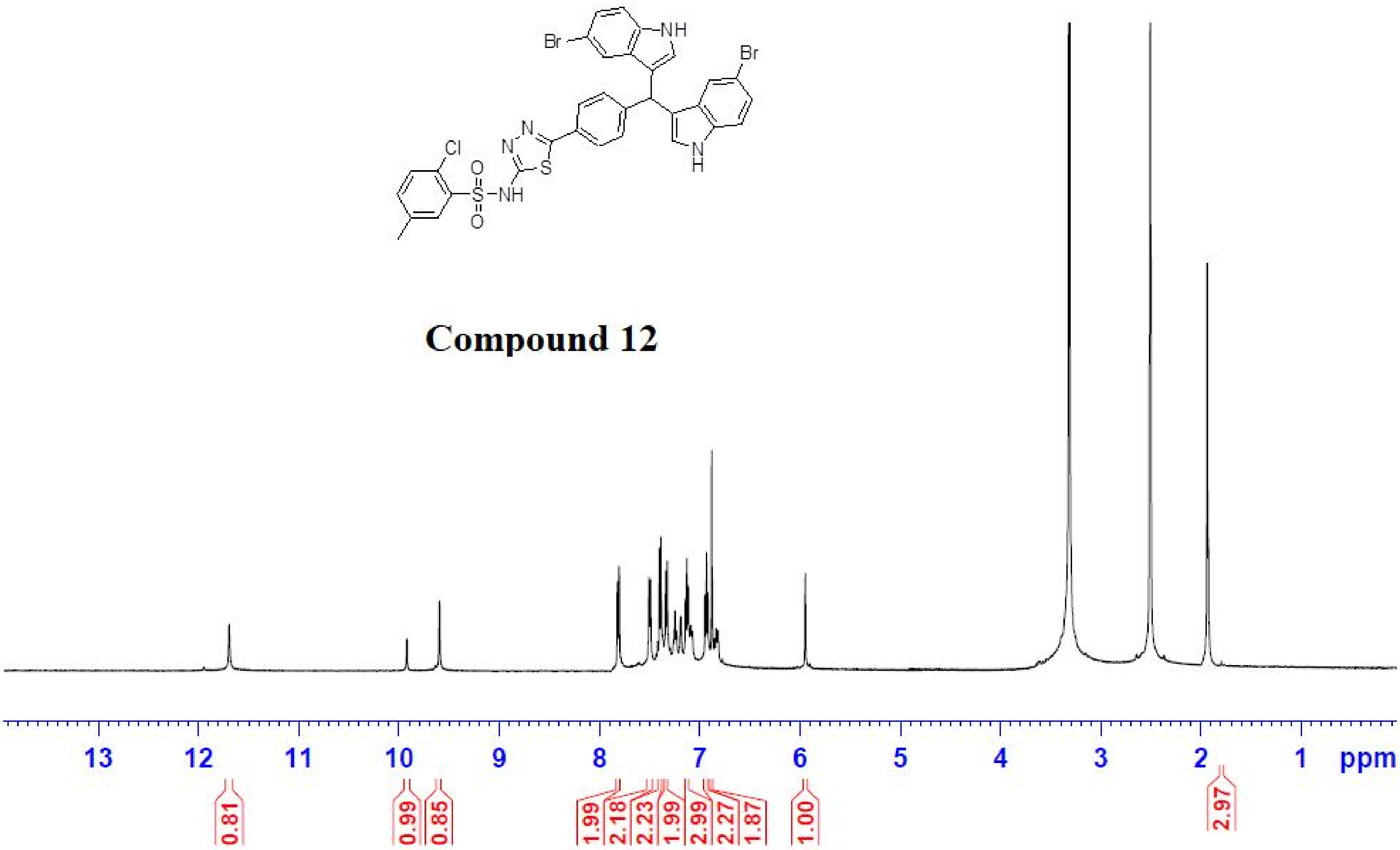
Br
NH
Br
N
N
Cl
O
S
NH
O
S
N
H
13
12
11
10
9
8
7
6
5
4
3
2
1
ppm
0.81
0.99
0.85
1.99
2.18
2.23
1.99
2.99
2.27
1.87
1.00
2.97

Compound 12

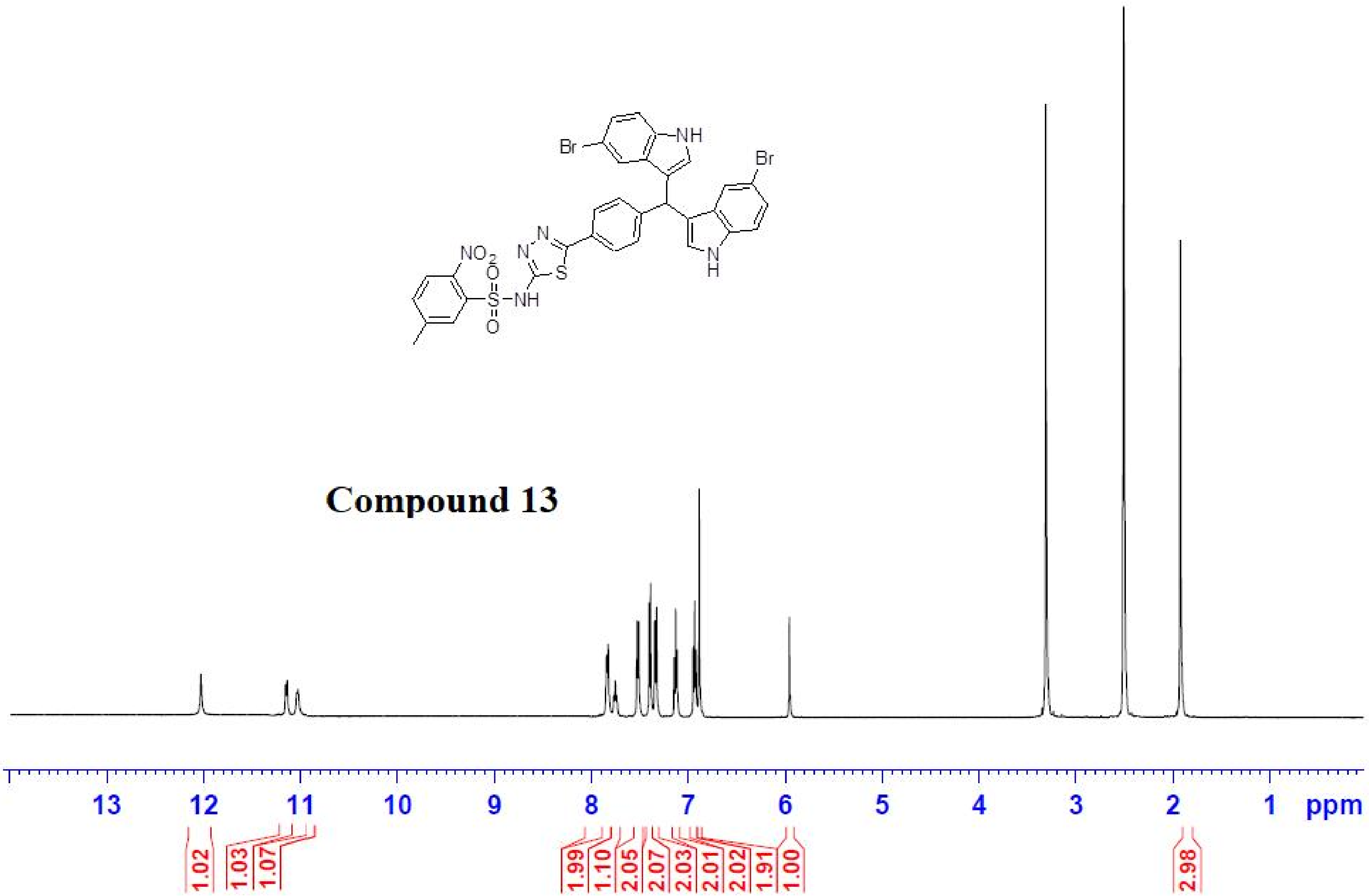
Compound 13
Br
NH
Br
N
H
NO2
N
N
O
S
S
NH
O
13
12
11
10
9
8
7
6
5
4
3
2
1
ppm
1.02
1.03
1.07
1.99
1.10
2.05
2.07
2.03
2.01
2.02
1.91
1.00
2.98

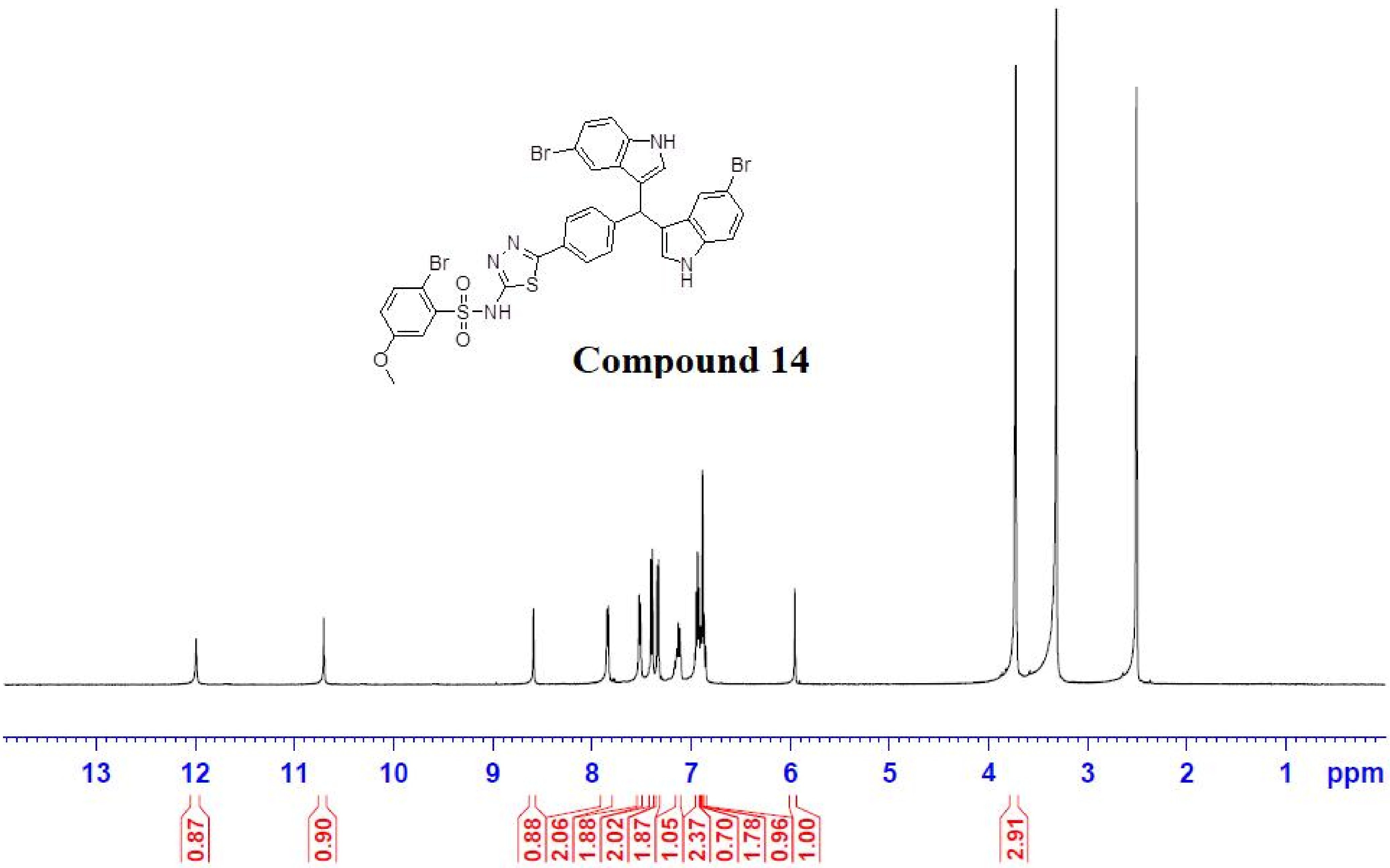
Br
NH
Br
N
N
Br
N
H
S
O
S
NH
O
O
Compound 14
13
12
11
10
9
8
7
6
5
4
3
2
1
ppm
0.87
0.90
0.88
2.06
1.88
2.02
1.87
1.05
2.37
0.70
1.78
0.96
1.00
2.91

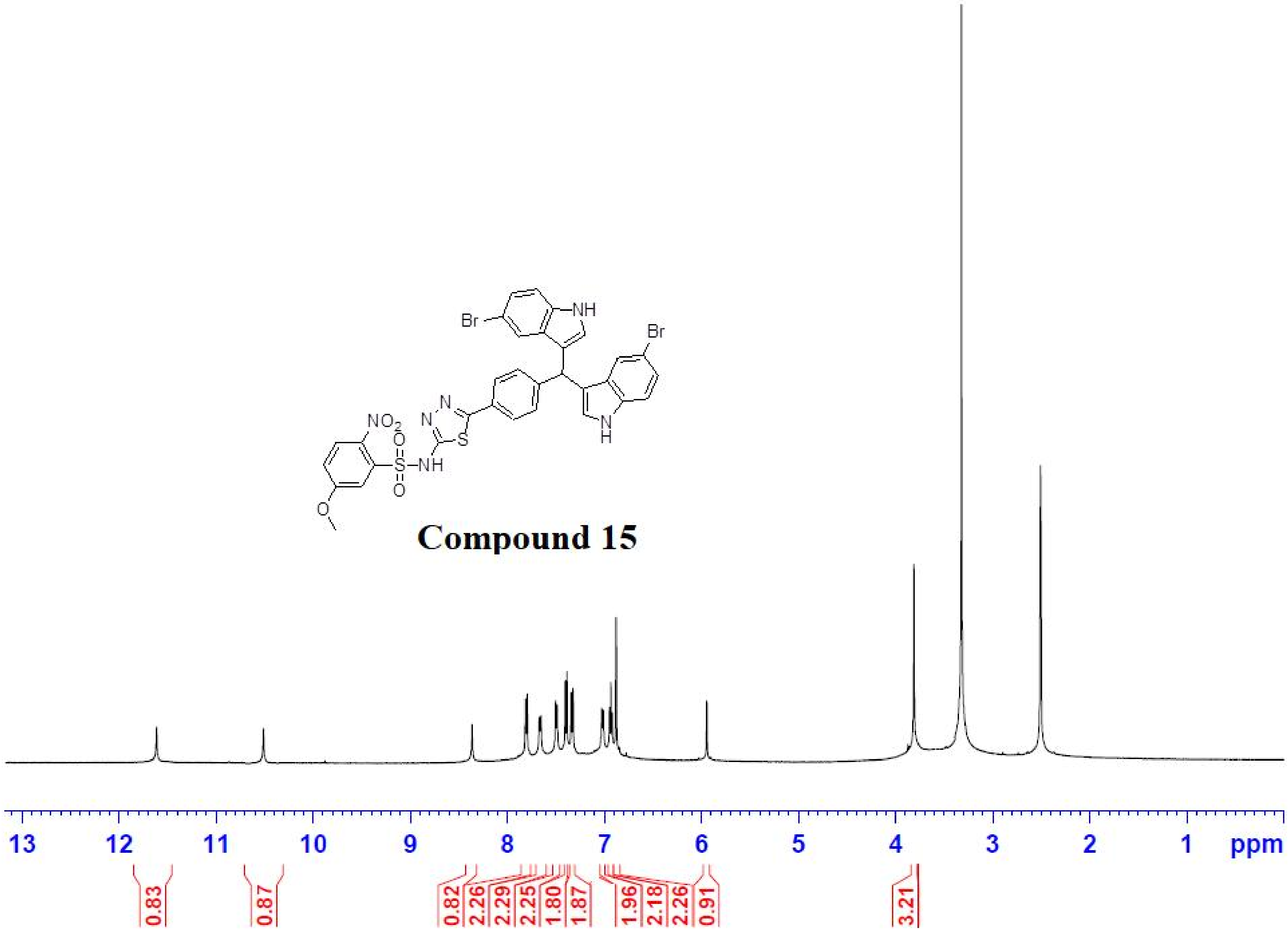
Br
NH
Br
N
N
NO2
O
S
O
NH
O
N
H
Compound 15
13
12
11
10
9
8
7
6
5
4
3
2
1
ppm
0.83
0.87
0.82
2.26
2.29
2.25
1.80
1.87
1.96
2.18
2.26
0.91
3.21

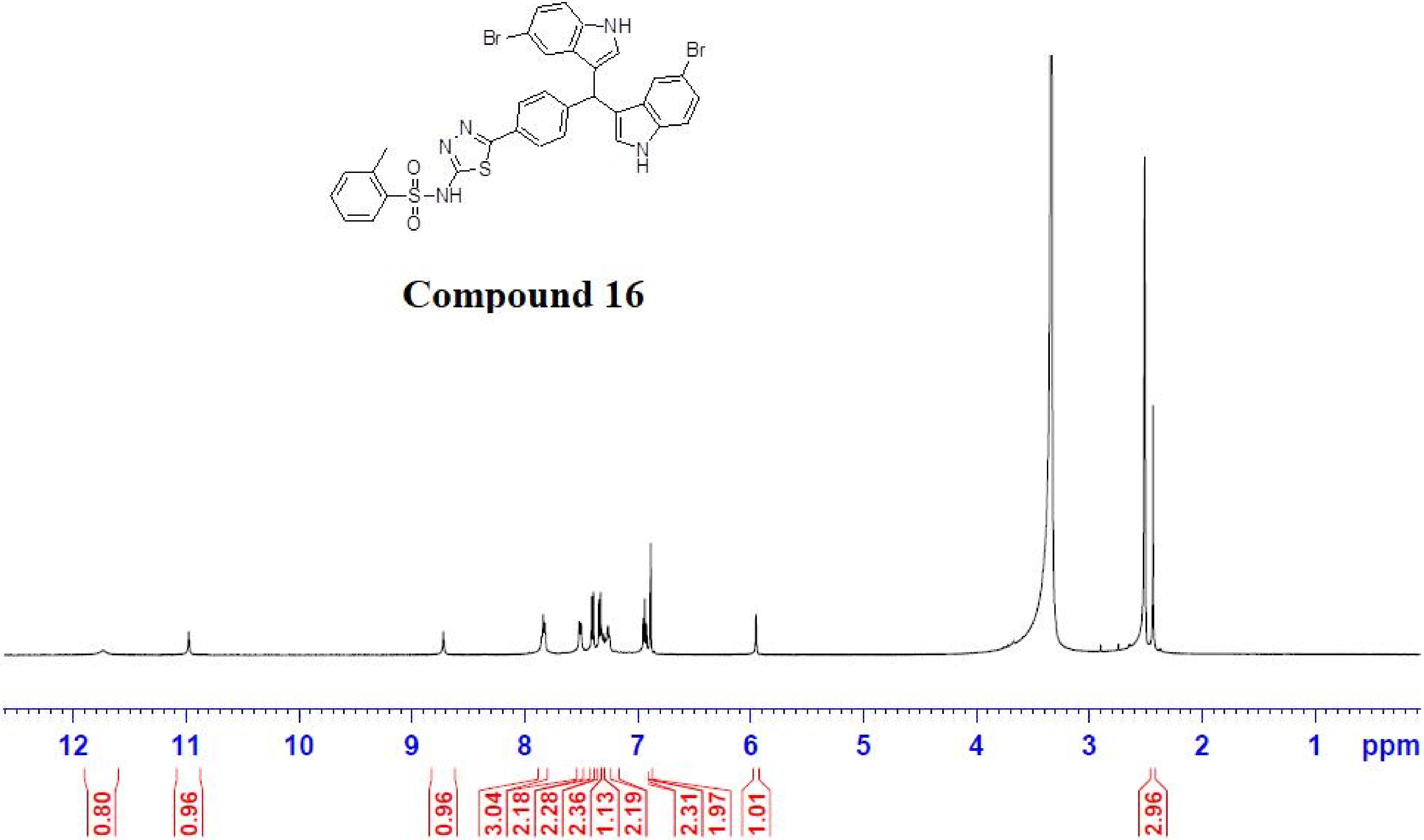
Br
NH
Br
N
N
S
O
S
NH
O
N
H
12
11
10
9
8
7
6
5
4
3
2
1
ppm
0.80
0.96
0.96
3.04
2.18
2.28
2.36
1.13
2.19
2.31
1.97
1.01
2.96

Compound 16

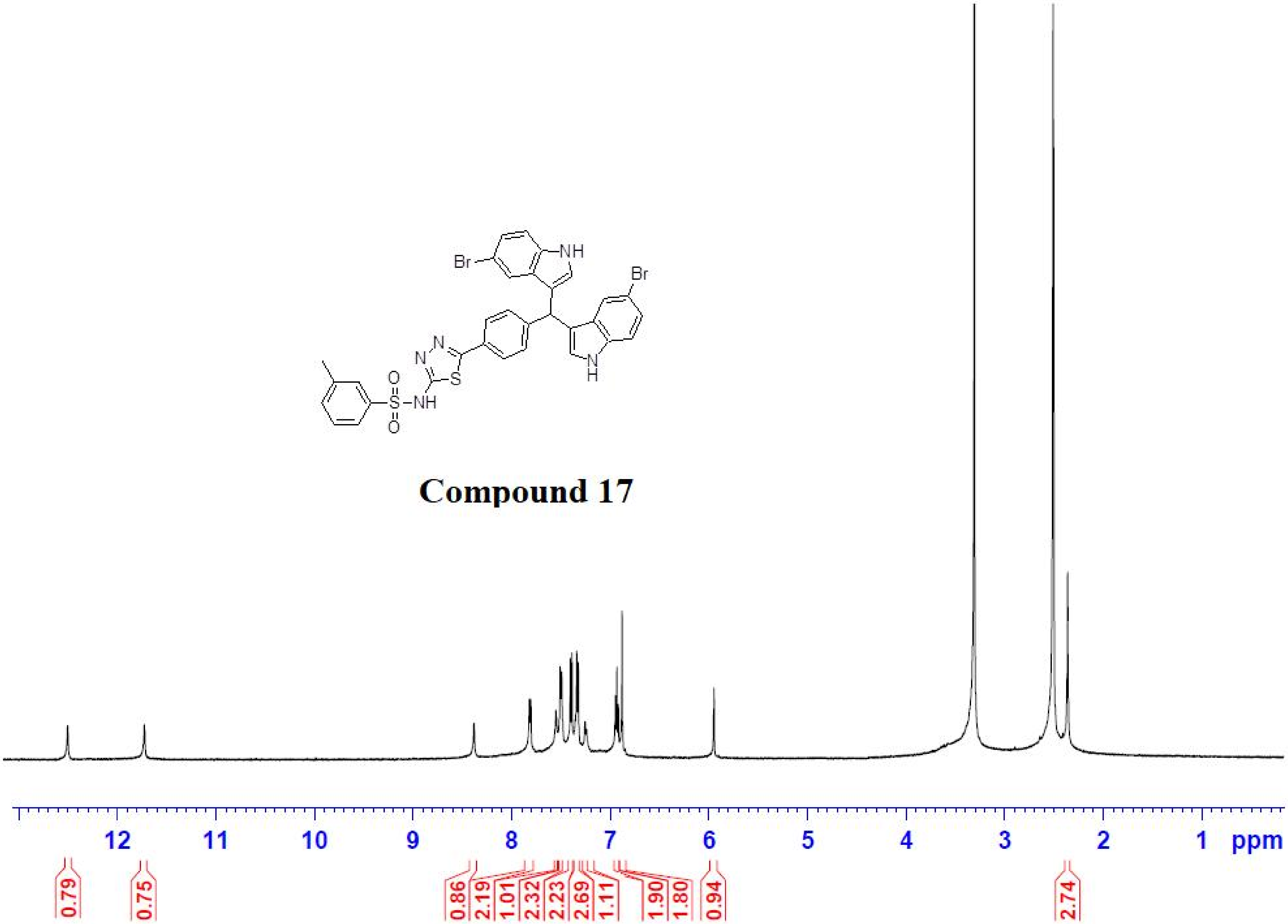
Br
NH
Br
N
N
N
H
O
S
S
NH
O
12
11
10
9
8
7
6
5
4
3
2
1
ppm
0.79
0.75
0.86
2.19
1.01
2.32
2.23
2.69
1.11
1.90
1.80
0.94
2.74

Compound 17

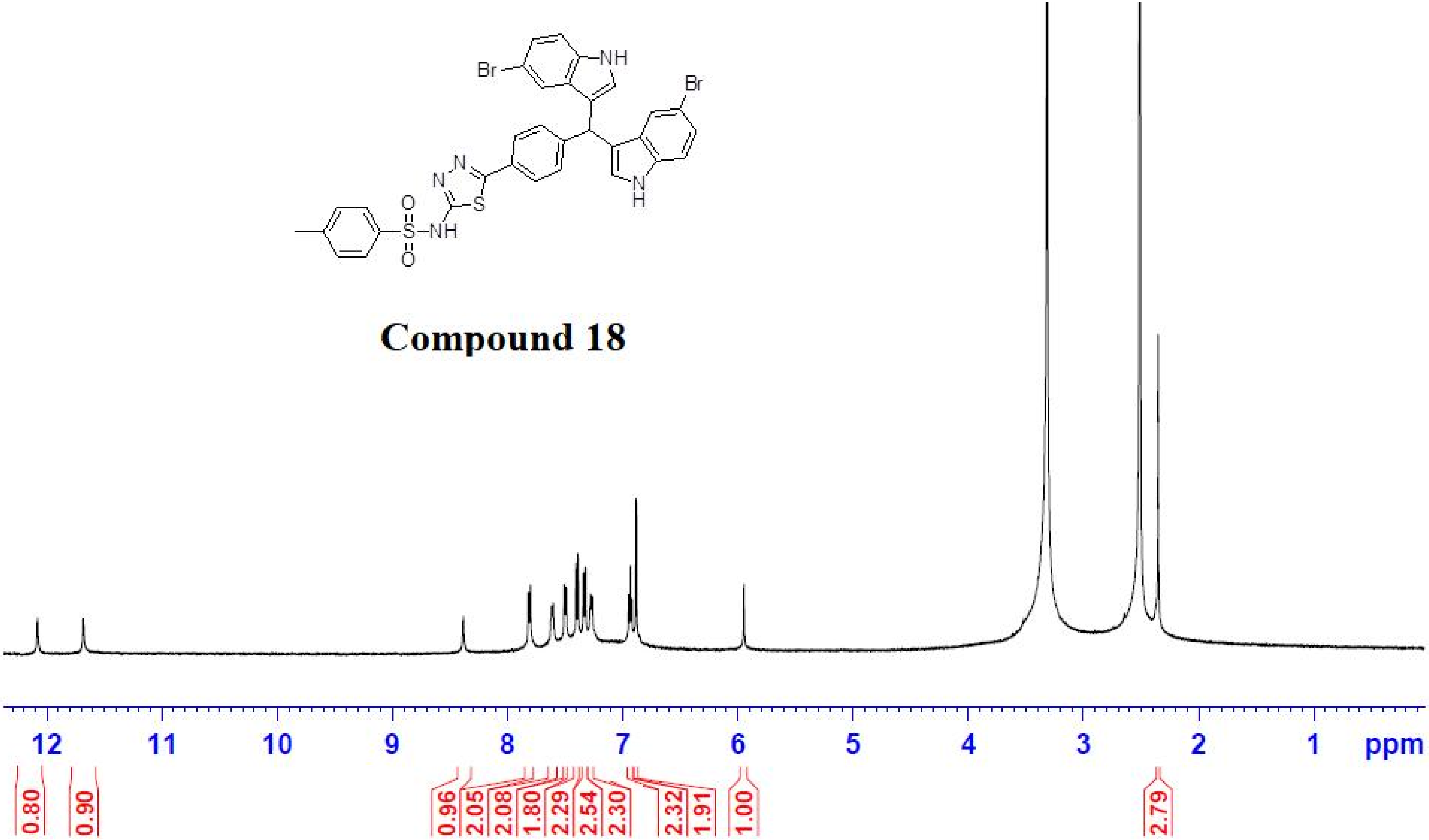
Br
NH
Br
N
N
N
S
O
S
NH
O
N
H
12
11
10
9
8
7
6
5
4
3
2
1
ppm
0.80
0.90
0.96
2.05
2.08
1.80
2.29
2.54
2.30
2.32
1.91
1.00
2.79

Compound 18
